# Effectiveness of Different Modalities of Lip Repositioning Surgery for Management of Patients Complaining of Excessive Gingival Display: A Systematic Review and Meta-Analysis

**DOI:** 10.1155/2021/9476013

**Published:** 2021-10-07

**Authors:** Shima Younespour, Siamak Yaghobee, Hoori Aslroosta, Neda Moslemi, Elham Pourheydar, Elaha Somaya Ghafary

**Affiliations:** ^1^Dentistry Research Institute, Tehran University of Medical Sciences, Tehran, Iran; ^2^Department of Periodontics, School of Dentistry, Tehran University of Medical Sciences, Tehran, Iran; ^3^School of Dentistry, Tehran University of Medical Sciences, Tehran, Iran; ^4^Department of Periodontics, School of Dentistry, Kabul University of Medical Science, Afghanistan

## Abstract

**Purpose:**

This study is aimed at synthesizing the available evidence regarding effectiveness of various modalities (combinations of LRS tasks) and comparison between each two modalities in terms of gingival display reduction, success rate, stability of the results, patient's satisfaction, and postoperative morbidity.

**Materials and Methods:**

The electronic databases including PubMed, Scopus, Web of Science Cochrane Library, Google Scholar databases, ClinicalTrials.gov, and WHO International Clinical Trial Registry Platform were searched up to 27th June 2020 regarding lip repositioning surgery. The modalities were defined as the combinations of the following tasks: frenectomy (yes/no), flap thickness (full/partial), and myotomy (yes/no). Meta-analyses were performed on gingival display change from baseline to months 3, 6, and 12 in each modalities using Stata (v.16).

**Results:**

38 studies (including three clinical trials, two quasiexperimental studies, seven case series, and 26 case reports) met the criteria for final inclusion. The mean gingival display reduced from baseline to 6 months (WMD = −2.90, 95% CI: -4.85 to -0.95) in the patients undergoing the “frenectomy + full-thickness flap + myotomy” modality. This parameter decreased from baseline to 6 and 12 months, respectively (WMD = −2.68, 95% CI: -3.49 to -1.86; WMD = −2.52, 95% CI: -4.40 to -0.64), in patients undergoing the “frenectomy + partial-thickness flap + without myotomy” modality. In patients who undergone the “without frenectomy + partial-thickness flap + without myotomy” modality, gingival display reduced from baseline to 6 months (WMD = −3.22, 95% CI: -5.61 to -0.84). Almost 83% of patients with modality 1 had satisfaction.

**Conclusions:**

Gingival display within the 6 months after LRS could be reduced with all modalities. Descriptively, the greatest reduction was observed in patients with the modality not including the frenulum.

## 1. Introduction

Smile is the most important facial expression that has a positive impact on the facial attractiveness and social interactions [1]. An ideal smile is based on a balance among three interrelated components: teeth, gingiva, and lips [2]. The exposure of more than 3 mm of maxillary gingiva has been considered as “unattractive smile,” “gummy smile,” or “excessive gingival display” (EGD) [3]. The prevalence of EGD in the 20- to 30-year-old US population has been estimated to be about 10%, and it was more prevalent in women than men [4]. While EGD has been regarded as an anatomic variation [5], there are an increasing number of patients seeking for correction of gummy smile. The results of a study by Malkinson et al. revealed that EGD influences negatively on the individual's perception of self-confidence, trustworthiness, attractiveness, intelligence, and friendliness [6]. The possible etiologic factors for EGD are based on skeletal, soft tissue, and dental discrepancies. The management of patients complaining of EGD is based on the etiology of this manifestation. Among the various treatment options, lip repositioning surgery (LRS) is utilized in a wide range of clinical situations with EGD and is the primary indication for mild-to-moderate vertical maxillary excess as well as excessive mobility of maxillary lip [7]. With the growing trend toward the use of less invasive treatment options, recently, LRS has been gaining popularity among the clinicians.

In the original technique, the frenulum was included in the surgical site; two partial-thickness elliptical incisions were made between the two projections of the labial commissures during smiling. The upper incision was at the buccal vestibular depth and the lower incision was at 2-3 mm above the dentoalveolar junction. The mucosa was then removed, and the upper wound edge was undermined and advanced. Then, it was sutured to the lower wound border with interrupted sutures [8]. It has been stated that this procedure has merit, and the plastic surgeons should be more widely familiar with this technique [9]. Later on, many modifications [9–13] have been proposed to improve the aesthetic outcome, to increase the stability of the results, and to reduce the risk of the postoperative complications. These modifications include frenulum sparing [12], full-thickness flap [9], and myotomy of the lip elevator muscles [11].

We found three relevant systematic reviews that were designed to answer some questions related to LRS. In a systematic review by Tawfik et al. in 2018, the gingival display reduction and the stability of the results were considered the outcomes. No related clinical trial had been published at that time point to be included in their systematic review [14]. Another systematic review in 2020 evaluated the short- and long-term gingival display reduction and focused on the comparison between LRS with and without myotomy [15]. Furthermore, the recently published systematic review in 2021 assessed the 6-month gingival display reduction in the patients who undergone LRS with or without myotomy [16]. Noteworthy, LRS comprises several important tasks including frenectomy (yes/no), flap thickness (full/partial), and myotomy (yes/no), and all these tasks can influence the outcomes of the surgery. Thus, grouping based on only one surgical task might induce confounding impacts on the results of LRS. To the best of our knowledge, this is the first study to evaluate the various combinations of lip repositioning surgical tasks (modalities) regarding clinical and patient-reported outcomes. Therefore, the current study is aimed at evaluating the effectiveness of different modalities of LRS in terms of gingival display reduction, success rate, stability of the results, patient's satisfaction, and postoperative morbidity.

## 2. Methods

This systematic review was done in line with the statement of preferred reporting items for systematic reviews and meta-analyses (PRISMA) [17]. This systematic review was registered in the International Prospective Register of Systematic Reviews (PROSPERO) (Number: CRD42020186234).

### 2.1. Focused Questions

*Q1*. Are various LRS modalities effective in the reduction of gingival display in patients with EGD?

*Q2*. What is the success rate of various modalities of LRS?

*Q3*. Are the results produced by different lip repositioning surgical modalities stable?

*Q4*. What is the frequency of patients with complete relapse in various modalities of LRS?

*Q5*. Are the patients satisfied with the results of different lip repositioning surgical modalities?

*Q6*. What is the rate of postoperative morbidities following various modalities of LRS in terms of lip tension, pain, or perioral numbness?

*Q7*. PICO: in patients with EGD, does any lip repositioning surgical modality improve the gingival display reduction, success of treatment, stability of the result, complete relapse, patient's satisfaction, and postoperative morbidity compared to another modality?

Lip repositioning surgical modalities were defined as follows:

Modality 1: LRS with frenectomy + full-thickness flap + with myotomy

Modality 2: LRS with frenectomy + partial-thickness flap + with myotomy

Modality 3: LRS with frenectomy + partial-thickness flap + without myotomy

Modality 4: LRS without frenectomy + full-thickness flap + with myotomy

Modality 5: LRS without frenectomy + partial-thickness flap + with myotomy

Modality 6: LRS without frenectomy + partial-thickness flap + without myotomy.

### 2.2. Inclusion and Exclusion Criteria

#### 2.2.1. Types of Studies

Randomized controlled trials (RCTs), controlled (nonrandomized) clinical trials, controlled before-after studies, quasiexperimental (nonrandomized) studies without control group, prospective and retrospective comparative cohort studies, case-control, case series, and case reports were included. Review articles, letters, animal studies, and editorials were excluded from the current study.

#### 2.2.2. Participants

We included studies focused on patients with EGD during smile, systemically healthy adult humans aged 18 years and above. No restriction was considered for either gender or ethnicity.

#### 2.2.3. Lip Repositioning Surgical Techniques

We enrolled all studies using any modality of LRS. Studies with a minimum follow-up period of 3 months that reported at least one of the outcomes were included. The studies in which the LRS was done as an adjunct to other surgical procedures such as crown lengthening or botulinum toxin injections were excluded.

#### 2.2.4. Types of Outcome Measures

*(1) Primary Outcomes*.

1. Gingival display change: gingival display change was defined as the change in the amount of gingival display (mm) from baseline to 3, 6, and 12 months after surgery. The gingival display was defined as the amount of gingival show during either active or passive smile
2. Success of treatment: if the amount of gingival display at 6 or 12 months after surgery was at most 3 mm, it was considered successful at that time point [3]
3. Stability of the results: if the amount of gingival display at 6 or 12 months after surgery was the same as that of obtained at 1 month, it was considered stable at that time point. Stability of LRS was considered only for studies with at least 6 months of follow-up
4. Complete relapse: if the gingival display at 6 or 12 months after surgery was the same as that of baseline, we defined it as complete relapse at that time point. Complete relapse was considered only for studies with at least 6 months of follow-up
5. Patient's satisfaction: the amount of patient's satisfaction with the LRS outcome.

*(2) Secondary Outcomes*.

1. Lip tension: the amount of upper lip stiffness during active smile in the first 3 weeks of postoperative follow-up
2. Postoperative pain: the amount of postoperative pain within three weeks after LRS
3. Postoperative numbness: the presence of postoperative numbness up to 3 weeks following LRS.

### 2.3. Information Sources and Search Strategy

Medline (through PubMed), Scopus, Embase, Web of Science, Cochrane Library, Google Scholar electronic databases, ClinicalTrials.gov, and WHO International Clinical Trial Registry Platform were searched up to 27th June 2020 without restrictions on publication year or language. Also, grey literature search was performed through open grey. Reference lists of included articles were manually screened. Four key journals were hand searched including the *Journal of Clinical Periodontology*, *Journal of Periodontology*, *Journal of Esthetic and Restorative Dentistry*, and *International Journal of Periodontics and Restorative Dentistry.*

Medical Subject Headings (MeSh) and Embase Subject Headings (Emtree) were used to find search terms. All relevant keywords were found by identifying word variants of keywords, synonyms, and related concepts used together with the Boolean operator “OR” for the search syntax. After finalizing the search syntax for PubMed, it was adapted to other databases. Search syntax for PubMed is reported in Table S1.

In addition, PROSPERO was searched for ongoing or recently completed systematic reviews.

### 2.4. Study Selection

First, all studies retrieved from electronic and manual searches were entered into EndNote X8.1, and the duplicates were removed. Two authors (NM and ESGh) reviewed the titles and abstracts of the studies, independently. For the articles with missing full-text, we contacted the authors through email and asked them to send the full-text. Furthermore, full-text screening for the remaining studies were performed by two reviewers (EP and ESGh) considering inclusion and exclusion criteria. All full-texts of the studies meeting the inclusion criteria were enrolled in the current study. Any disagreement on certain studies was resolved discussing with an expert third person (SY).

### 2.5. Data Extraction

The following information were extracted from the included studies by two reviewers (ShY, ESGh), separately: authors' name, year of publication, type of study, country, age, gender, etiology, details of lip repositioning surgical technique, type of instrument used for incision (laser or scalpel), gingival display at baseline and follow-up time points (1st, 3rd, 6th, and 12th months), complete relapse at 6 and 12 months after surgery, stability of the results at 6 and 12 months posttreatment, treatment success at 6 and 12 months after surgery, patient's satisfaction, postoperative morbidities (lip tension, pain, and perioral numbness), follow-up period, and any comment.

### 2.6. Risk of Bias Assessment

Two blinded reviewers (ESGh, ShY) assessed the quality of the included studies, independently. Any disagreement was resolved by discussion with a third reviewer (SY). The quality of the clinical trials was determined using the Cochrane Handbook for Systematic Reviews of Interventions Guidelines [18] and the CONSORT statement [19]. The total judgment was as follows: low risk of bias (if all the domains were considered low risk of bias); unclear risk of bias (if at least one item was judged as unclear risk of bias); or high risk of bias (if at least one item was considered high risk of bias). For the assessment of case reports, case series, and quasiexperimental studies without control group, we used the Joanna Briggs Institute (JBI) Critical Appraisal Checklist tool. The JBI has separate checklists for quasiexperimental studies (9 questions) [20], case series (10 questions) [21], and case reports (8 questions) [22]. The total scores of 0-4 were considered high risk of bias for quasiexperimental studies and case series. For case reports, the total scores of 0-3 were considered high risk of bias.

### 2.7. Statistical Methods

The statistical analyses were performed by the biostatistician (ShY). The primary and secondary outcomes were summarized as follows: number (percent) for nominal variables, mean ± SD, and range for continuous variables. The mean gingival display change from baseline to endpoint was computed as the mean gingival display at endpoint minus mean gingival display at baseline. Review Manager (RevMan) (computer program version 5.4, The Cochrane Collaboration, 2020) was used for graphical overview of risk of bias in the included studies.

Meta-analyses were conducted on gingival display change from baseline to endpoint (months 3, 6, and 12) in each LRS modalities. These analyses were done using StataCorp 2019 (Stata Statistical Software: Release 16; College Station, TX: StataCorp LLC). Studies with at least ten patients were included in the meta-analyses. These meta-analyses were planned to answer the first question of the current systematic review. The implementation of a random effects model was considered more appropriate based on the diversity of study designs and patient characteristics.

## 3. Results

### 3.1. Study Selection

**Figure 1:**
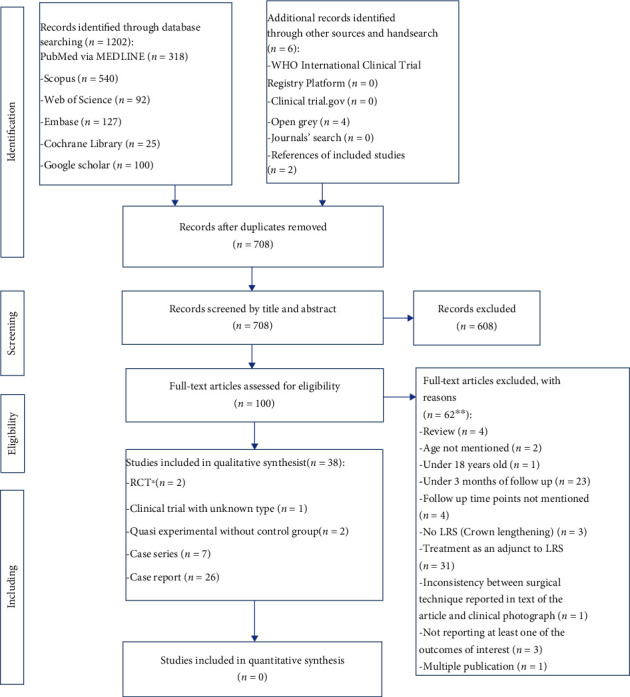
PRISMA study flow diagram. ^∗^One of the RCTs was a randomized clinical trial with two arms including modified lip repositioning surgery (LRS) and nonsurgical technique using Botulinum toxin type-A injection. According to our inclusion criteria, only the LRS group was included in our study. ^∗∗^There were some studies with more than one reason for exclusion.

The study flow diagram is presented in Figure 1. Initially, the search strategy retrieved 1208 studies. After removing duplicates, 708 records remained. After screening the titles and abstracts, 608 articles were excluded due to unrelated topic or having adjunctive treatment. The full-texts of the remaining 100 articles were assessed, and 38 articles met the prespecified inclusion criteria and were therefore included in the current study. Characteristics of excluded studies are presented in Table S2.

### 3.2. General Characteristics of the Included Studies

The 38 included studies assessed different modalities of LRS in 160 patients. Two RCTs [23, 24] one clinical trial (with unknown type) [25], two quasiexperimental studies without control group [12, 26], seven case series [27–33], and 26 case reports [10, 34–58] met the inclusion criteria. For the one RCT with two arms of LRS and botulinum toxin type-A injection, we included the LRS arm in our study [24]. Characteristics of the included studies are reported in Table S3(a, b, c, and d).

Out of 38 articles included in this review, 36 were in English language. Two articles in Japanese [56] and Korean [51] languages were translated in English.

The included studies have been published from 2006 to 2020. Follow-up period of the studies ranged from three months to four years. We contacted the authors of 36 studies in an effort to obtain additional information. Twelve authors responded to our emails [12, 23, 24, 28, 32, 39–41, 52–54, 57]. Obtained data from five authors was added to the tables [28, 32, 39, 41, 52, 54].

### 3.3. Characteristics of the Participants

Three clinical trials included 82 individuals (76 females and 6 males) and aged 18 to 38 years old [23–25]. Two quasiexperimental studies had 29 patients (27 females and 2 males), aged 19 to 49 years old, with 4 to 10 mm gingival display [12, 26]. Seven case series reported 43 patients aged 18 to 59 years old and 4.73 to 8 mm gingival display [27–33]. There were 25 females and 2 males in 27 included case reports from 18 to 38 years old with EGD during dynamic smile Table S3 (a, b, c, and d) [10, 34–58].

### 3.4. Risk of Bias in Included Studies

**Figure 2:**
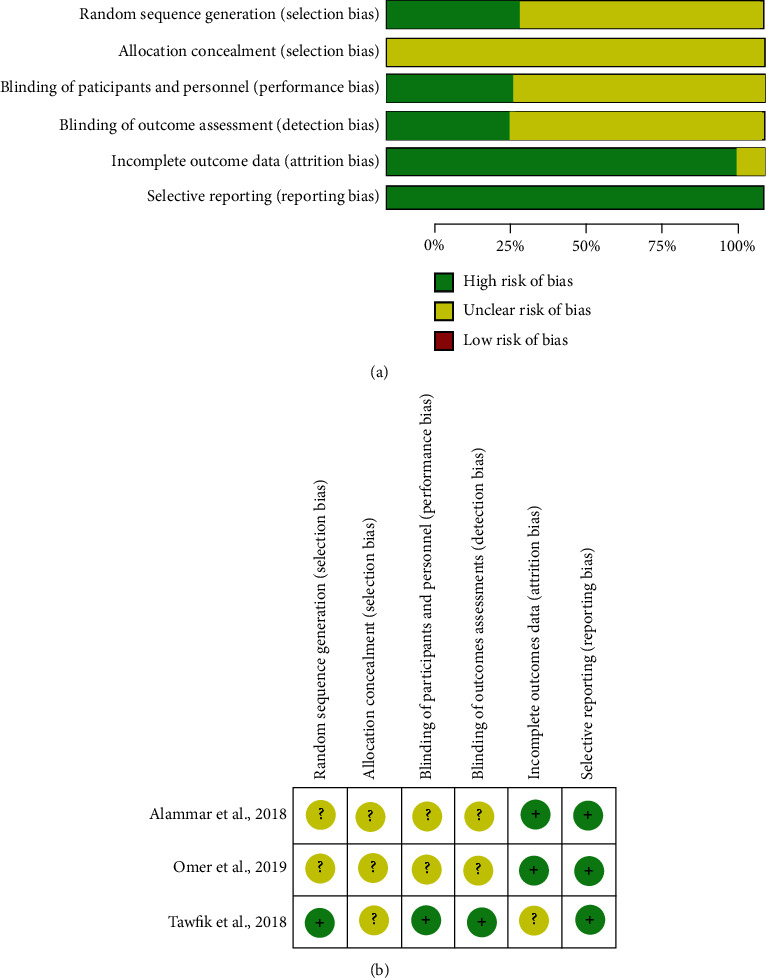
(a) Risk of bias graph: review authors' judgments about each risk of bias item presented as percentages across all included studies. The graph is drawn by Review Manager (RevMan) (computer program, version 5.4, The Cochrane Collaboration, 2020). (b) Risk of bias summary: review authors' judgments about each risk of bias item for each included study. The graph is drawn by Review Manager (RevMan) (computer program, version 5.4, The Cochrane Collaboration, 2020).

Risk of bias in the included clinical trials as a graphical overview is illustrated in Figure 2(a). Reviewed authors' judgments about each risk of bias item for each included clinical trial are summarized in Figure 2(b). The total judgment was “unclear risk of bias” for all three clinical trials [23–25]. None of the included quasiexperimental studies were found to be at high risk of bias (tables S4 (a)) [12, 26]. The total scores of JBI checklists for critical appraisal ranged from 3 to 8 for case series with two studies at high risk of bias ((tables S4(b)) [28, 32] and ranged from 3 to 8 for case reports with one article considered at high risk of bias (tables S4(c) [50].

### 3.5. Summary of Included Studies and Meta-Analyses

Summary of primary and secondary outcomes in each of the lip repositioning surgical modalities and study types is presented in Table 1. Focused questions are answered as follows:

### 3.6. Q1: Are Various LRS Modalities Effective in the Reduction of Gingival Display in Patients with EGD?

#### 3.6.1. Modality 1: LRS with Frenectomy + Full-Thickness Flap + with Myotomy

**Figure 3:**
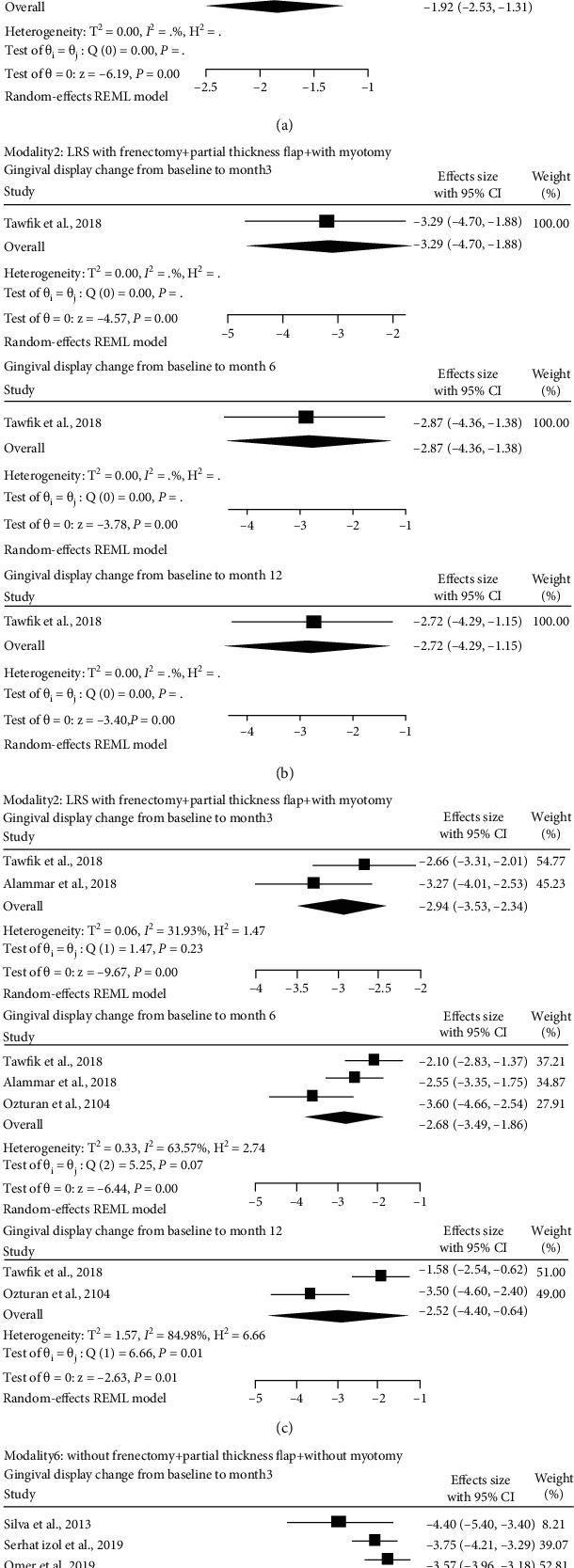
(a) Forest plot of estimated mean gingival display change from baseline to months 3, 6, and 12 after lip repositioning surgical modality 1 (LRS with frenectomy + full-thickness flap + with myotomy). (b) Forest plot of estimated mean gingival display change from baseline to months 3, 6, and 12 after lip repositioning surgical modality 2 (LRS with frenectomy + partial-thickness flap + with myotomy). (c) Forest plot of estimated mean gingival display change from baseline to months 3, 6, and 12 after lip repositioning surgical modality 3 (LRS with frenectomy + partial-thickness flap + without myotomy). (d) Forest plot of estimated mean gingival display change from baseline to months 3, 6, and 12 after lip repositioning surgical modality 6 (LRS without frenectomy + partial-thickness flap + without myotomy).

The mean gingival display decreased significantly from baseline to 3 and 6 months, respectively: WMD = −2.98 mm, 95% CI: -5.10 to -0.85, *n* = 23, and WMD = −2.90 mm, 95% CI: -4.85 to -0.95, *n* = 23 (Figure 3(a)) [25, 27]. However, heterogeneity between studies was observed as presented in Figure 3(a). According to one case series, the mean gingival display reduced significantly from baseline to 12 months (MD = −1.92 mm, 95% CI: -2.53 to -1.31; *n* = 12; Figure 3(a)) [27].

#### 3.6.2. Modality 2: LRS with Frenectomy + Partial-Thickness Flap + with Myotomy

According to one clinical trial (10 patients; one arm), the mean gingival display reduction from baseline to 3, 6, and 12 months was as follows, respectively: MD = −3.29 mm, 95% CI: -4.70 to -1.88; MD = −2.87 mm, 95% CI: -4.36 to -1.38; and MD = −2.72 mm, 95% CI: -4.29 to -1.15 (Figure 3(b)) [23].

#### 3.6.3. Modality 3: LRS with Frenectomy + Partial-Thickness Flap + without Myotomy

The mean gingival display reduction from baseline to 3 and 6 months was as follows, respectively: WMD = −2.94 mm, 95% CI: -3.53 to -2.34, *n* = 21 (two clinical trials) [23, 25], and WMD = −2.68 mm, 95% CI: -3.49 to -1.86; *n* = 31 (two clinical trials and one case series) [23, 25, 29] (Figure 3(c)). The mean gingival display decreased significantly from baseline to 12 months (WMD = −2.52 mm, 95% CI: -4.40 to -0.64; *n* = 20; one clinical trial and one case series) [23, 29]. However, heterogeneity between studies was found as presented in (Figure 3(c)).

#### 3.6.4. Modalities 4 and 5

No data available.

#### 3.6.5. Modality 6: LRS without Frenectomy + Partial-Thickness Flap + without Myotomy

The mean gingival display decreased significantly from baseline to 3 months (WMD = −3.71 mm, 95% CI: -3.99 to -3.42; *n* = 49; one clinical trial and two quasiexperimental groups, Figure 3(d)) [12, 24, 26]. The mean gingival display reduced significantly from baseline to 6 months (WMD = −3.22 mm, 95% CI: -5.61 to -0.84; *n* = 29; two quasiexperimental groups) [12, 26]. However, heterogeneity between studies was observed as presented in Figure 3(d). The pattern of gingival display change differed between the two quasiexperimental studies during six months of follow-up (Table S3(b)) [12, 26]. There was no study with 12-month follow-up period after surgery to compute mean gingival change from baseline to 12 months.

### 3.7. Q2-Q6

The results of primary and secondary outcomes in different modalities of LRS are summarized in Table 1.

### 3.8. Q7: PICO: In Patients with EGD, Does Any Lip Repositioning Surgical Modality Improve the Gingival Display Reduction, Success of Treatment, Stability of the Result, Complete Relapse, Patient's Satisfaction, and Postoperative Morbidity Compared to Another Modality?

#### 3.8.1. Modality 2 (LRS with Frenectomy + Partial-Thickness Flap + with Myotomy) vs. Modality 3 (LRS with Frenectomy + Partial-Thickness Flap + without Myotomy)

**Figure 4:**
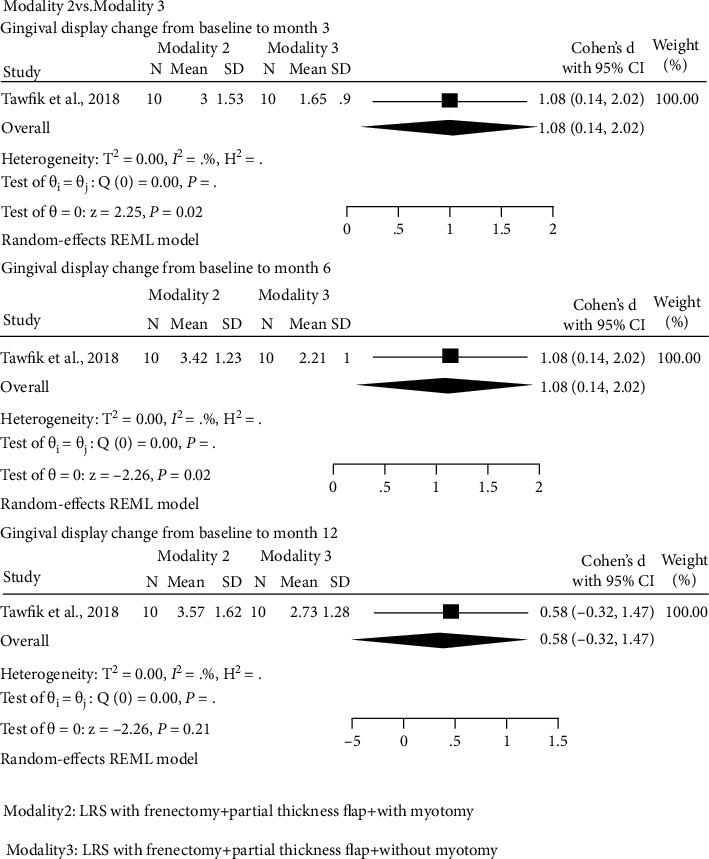
Forest plot of the effect size for comparative study (modality 2 vs. modality 3) at 3, 6, and 12 months after surgery. Modality 2: LRS with frenectomy + partial-thickness flap + with myotomy. Modality 3: LRS with frenectomy + partial-thickness flap + without myotomy.

There was one study to compare between modality 2 and modality 3 [23]. In Tawfik's study, there were no sufficient data to calculate standard error of effect size from the pretest-posttest-control design [59]. The mean gingival display was significantly higher in patients who undergone modality 2 in comparison with modality 3 at baseline (SMD: 0.98, 95% CI: 0.05 to 1.91), at month 3 (SMD: 1.08, 95% CI: 0.14 to 2.01), and at month 6 (SMD: 1.08, 95% CI: 0.14 to 2.02). There was no significant difference between these two modalities in month 12 after surgery (SMD: 0.58, 95% CI: -0.32 to 2.01; Figure 4). The results of other outcomes are summarized in Table S3(a).

#### 3.8.2. Modality 1 (LRS with Frenectomy + Full-Thickness Flap + with Myotomy) vs. Modality 3 (LRS with Frenectomy + Partial-Thickness Flap + without Myotomy)

**Figure 5:**
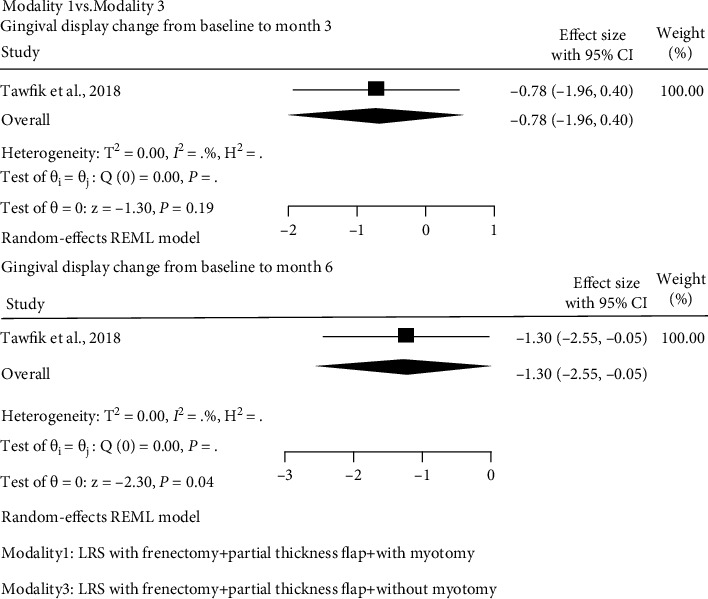
Forest plot of the effect size for modality 1 compared to modality 3 on mean gingival display reduction from baseline to 3 and 6 months. Modality 1: LRS with frenectomy + full-thickness flap + with myotomy. Modality 3: LRS with frenectomy + partial-thickness flap + without myotomy.

One study was found to compare between modality 1 and modality 3 [25]. In this study, there were sufficient data to estimate the effect size from the pretest-posttest-control design [59]. Modality 1 in comparison with modality 3 did not differ significantly in mean gingival display reduction from baseline to month 3 posttreatment (SMD: -0.78, 95% CI: -1.96 to 0.40; Figure 5). However, additional decrease in mean gingival display was observed from baseline to 6 months posttreatment with modality 1 compared to modality 3 (SMD: -1.30, 95% CI: -2.55 to -0.05; Figure 5). The results of other outcomes are presented in Table S3(a).

#### 3.8.3. Other Comparisons

No studies available.

## 4. Discussion

The present systematic review and meta-analysis were conducted to assess the effectiveness of various lip repositioning surgical modalities in the treatment of EGD patients. Each modality of LRS comprises several important tasks including frenectomy (yes/no), flap thickness (full/partial), and myotomy (yes/no) in which the outcomes of the surgery can be influenced by these tasks. Thus, grouping based on only one surgical task might induce confounding impacts on clinical and patient-reported outcomes. To avoid encountering substantial heterogeneity among studies, the current study is aimed at evaluating various modalities of LRS and comparing them with each other. The previous systematic reviews did not consider this important issue [14–16].

We have classified the lip repositioning surgical procedures into 6 modalities, based on the practical point of view. The most frequently used modality was modality 3 which was the original technique introduced by Kostianovsky and Rubinstein [8]. Other modalities used in the included studies were modalities 1, 2, and 6. No study was found to spare the midline frenulum while cutting the muscles (modalities 4 and 5), since these two modalities might not be technically feasible.

Case series and case reports were the most retrieved articles, and there were a limited number of well-designed studies. Some of the case series and most of the case reports reported subjective gingival display reduction without an exact measurement of pre- or postoperative gingival display [10, 30, 32, 34, 36, 39, 40, 42, 44, 47, 49, 53, 54, 58]. In the current study, there is lack of sufficient evidence in each modality in order to obtain conclusive results about the gingival display change from baseline to 3, 6, and 12 months after surgery. In addition, heterogeneity was found among studies. However, due to the low number of included studies, it was impossible to conduct subgroup analysis or meta-regression to find the source of heterogeneity. For the 6-month results, two articles with modality 1 [25, 27], three with modality 3 [23, 25, 29], and two with modality 6 [12, 26] were included in the meta-analyses. The results of the meta-analyses showed that all modalities could reduce the gingival display within the 6 months after surgery. On average, this reduction ranged from 2.68 mm to 3.22 mm in various modalities. Descriptively, the greatest gingival display reduction was associated with the modality which did not include the frenulum (modality 6). However, due to the lack of strong evidence, at present, it is not possible to draw conclusive results for comparison between each two modalities. For the 12-month results, two articles with modality 3 were considered included in the meta-analysis which showed 2.52 mm reduction in gingival display [23, 29].

As expected, the overall findings of the current systematic review and meta-analysis were consistent with previous meta-analyses. However, those studies did not focus on the modalities [14–16]. In our study, the amount of gingival display reduction differed in various modalities. Furthermore, we excluded those studies with adjunctive treatments to LRS; however, this issue was not considered in the previous meta-analyses [14–16].

The majority of the included studies focused on the results of LRS according to the amount of gingival display reduction. Since the candidate patients for LRS usually seek for a slight gingival exposure and ask about the success rate of this procedure, it seems that reporting the data in terms of the success rate of LRS needs to be considered in the studies. At present, there is not any established cut-off point between acceptable and unacceptable gingival display, as the amount of desired gingival display could be varied in different population and cultures. However, the results of an investigation demonstrated that the gingival exposure within 3 mm is esthetically accepted by the clinicians and laypeople [3]. We used the threshold of 3 mm postoperative gingival display in the current study to evaluate the success rate. According to the 6-month results, success of treatment has not been reported in 11 (48%) patients who undergone modality 1, all patients with modality 2, 66 (98%) patients with modality 3, and 43 patients (74%) patients with modality 6. Thus, due to the lack of data, it was not possible to conclude about the success rate of LRS in each modality and to compare between each two modalities. None of the previous systematic reviews considered the success rate of treatment as an outcome [14–16].

The risk of relapse after LRS has been concerned from the introduction of this procedure. However, we did not find any established definition for complete relapse or stability of the results for each patient who undergone LRS. For a number of studies, the stability of the results was not defined by the authors; nevertheless, in the result section, the treatment outcome was reported as stable [12, 36, 39, 40, 42, 46, 51–56, 58]. Therefore, stability and complete relapse outcomes in the current study were defined based on the judgment of the experts (NM and SY). Based on these definitions, there was only one case series which presented raw data for gingival display; so, we could describe these outcomes. This case series included 12 patients who undergone modality 1 [27]. Results showed that in 8 out of 12 patients (66.67%), the results obtained at one month remained stable after 6 and 12 months, and there was one patient with complete relapse at these time points [27]. The rationale for the occurrence of relapse is considered the presence of tension of muscle attachments during suturing. Therefore, LRS with myectomy/myotomy has been proposed to detach the smile muscle attachment and preclude the relapse. However, the method of myectomy/myotomy varies among studies [11, 23, 25, 27]. In the original method introduced by Miskinyar at 1983, the levator labii superioris muscles were removed about 1-2 cm. Briefly, two separate incisions with a width of 2 cm were made at the level of upper canine teeth. After the elevation of a full-thickness flap, these muscles were exposed and dissected carefully with a blunt instrument. The muscles were then amputated cautiously at the level of junction with orbicularis oris [11]. Although the method introduced by Miskinyar [11] is more invasive, resection of major muscles responsible for elevating the lip, levator labii superioris, seems to be mandatory for a successful result. However, it has not been mentioned in the latter studies [23, 25, 27]. Other methods have been used to prevent the risk of relapse are advancing the flap to remove the flap tension [36, 39, 40, 58] and using periosteal fenestration and extraoral tissue stabilization tapes to accelerate the process of scar formation during healing phase [31].

Due to the lack of evidence as mentioned by previous systematic review [14], we could not conclude about the stability and complete relapse outcomes in each modality of LRS and comparing between each two modalities. In addition, the number of studies with a long follow-up period (more than one year) was limited. Those in which followed the patients with more than 12 months did not report the gingival display [49, 52].

Patient's satisfaction with the treatment outcome is considered the key factor in determining the success of each treatment, especially in a procedure like LRS where esthetics is the main concern to the patients. However, satisfaction status has not been reported in 65 out of 160 patients (40.62%) treated with LRS. Nineteen out of 23 patients (82.61%) with modality 1 had satisfaction with LRS outcome [25, 27]. However, we could not conclude about the patient's satisfaction in the other modalities due to the high rate of missing data. Previous systematic reviews did not consider this outcome [14–16].

Postoperative morbidities were not reported in most studies as follows: 103 out of 160 patients (64.38%) for lip tension, 123 out of 160 patients (76.88%) for pain, and 77 out of 160 patients (48.12%) for perioral numbness. Furthermore, no study was found to report any patient complaining of lip tension in the long run. On the other hand, those studies addressing the postoperative pain reported that LRS was associated with mild pain during the first three weeks after surgery, irrespective of the type of lip repositioning surgical modality. None of the previous systematic reviews reported these morbidities [14–16].

### 4.1. Limitations

The results of the current systematic review and meta-analysis have to be interpreted cautiously with a number of limitations. Some limitations are as follows:

1. There were no or limited number of well-designed RCTs to compare between each two modalities of LRS. Including different study designs is a limitation of the current systematic review. To overcome the insufficiency of RCTs, other study designs were considered in the current study as well. However, the robustness of the results would be increased by the inclusion of only RCTs
2. There were incomplete data reported in the published primary studies
3. Subjective EGD improvement was reported by most of primary studies without an exact measurement of gingival display at pre- or postoperative treatment
4. There was a lack of standardized definitions of complete relapse, stability and success rate of treatment
5. There was a lack of studies showing the long-term (more than one year) effects of LRS on stability and success
6. Most primary studies came from Asian countries. Probably EGD is less prevalent in some regions and races
7. The included three clinical trials were judged as “unclear risk of bias.”

### 4.2. Suggestions

We suggest designing further primary studies with abovementioned modalities with adequate sample size, studies with high levels of evidence, and long-term follow-up. Furthermore, our recommendations for future studies are as follows: reporting all important outcomes of LRS with standardized definitions and objective measurements. In addition, it is suggested for future studies to evaluate if the position of the lower incision line in relation to the mucogingival junction, the lateral extension of the incision lines, and the distance between the two incisions have any influence on the clinical outcomes.

## 5. Conclusions

1. Meta-analyses in the present study showed that the gingival display within the 6 months after surgery could be reduced in all modalities
2. Descriptively, the modality which did not include the frenulum had the greatest gingival display reduction
3. Due to the lack of data and established definitions, it was not applicable to draw conclusive results about the success rate, complete relapse, and stability of LRS
4. Almost 83% of patients with “frenectomy + full-thickness flap + myotomy” modality had satisfaction with the LRS outcome
5. Those studies addressing the postoperative pain mentioned mild pain during the first three weeks after LRS.

## Figures and Tables

**Table 1:** Summary of the primary and secondary outcomes in each of the lip repositioning surgical modalities.

Outcomes in each type of study	Lip repositioning surgical modalities					
	Modality 1: LRS with frenectomy + full thickness + with myotomy	Modality 2: LRS with frenectomy + partial thickness + with myotomy	Modality 3: LRS with frenectomy + partial thickness + without myotomy	Modality 4: LRS without frenectomy + full thickness + with myotomy	Modality 5: LRS without frenectomy + partial thickness + with myotomy	Modality 6: LRS without frenectomy + partial thickness + without myotomy
^∗^Clinical trials: *N* = 3, *n* = 62	*N* = 1, *n* = 11	*N* = 1, *n* = 10	*N* = 2, *n* = 21	*N* = 0, *n* = 0	*N* = 0, *n* = 0	*N* = 1, *n* = 20
Mean GD change from baseline to 3rd month, mm	*N* = 1, *n* = 11 MD: -4.09 95% CI: -4.99 to -3.19	*N* = 1, *n* = 10 MD: -3.29 95% CI: -4.70 to -1.88	*N* = 2, *n* = 21 WMD: -2.94 95% CI: -3.53 to -2.34	—	—	*N* = 1, *n* = 20 ^#^MD: -3.57 95% CI: -3.96 to -3.18
Mean GD change from baseline to 6th month	*N* = 1, *n* = 11 MD: -3.91 95% CI: -4.64 to -3.18	*N* = 1, *n* = 10 MD: -2.87 95% CI: -4.36 to -1.38	*N* = 2, *n* = 21 WMD: -2.30 95% CI: -2.84 to -1.76	—	—	N/A (follow-up less than 6 months)
Mean GD change from baseline to 12th month	N/A (follow-up less than 12 months)	*N* = 1, *n* = 10 MD: -2.72 95% CI: -4.29 to -1.15	*N* = 1, *n* = 10 MD: -1.58 95% CI: -2.54 to -0.62	—	—	N/A (follow-up less than 12 months)
Success of treatment at 6 months after surgery	N/A: *n* = 11	N/A: *n* = 10	N/A: *n* = 21	—	—	N/A: *n* = 20
Success of treatment at 12 months after surgery	N/A: *n* = 11	N/A: *n* = 10	N/A: *n* = 10	—	—	N/A: *n* = 20
Stability at 6 months after surgery	N/A: *n* = 11	N/A: *n* = 10	N/A: *n* = 21	—	—	N/A: *n* = 20
Stability at 12 months after surgery	N/A: *n* = 11	N/A: *n* = 10	N/A: *n* = 10	—	—	N/A: *n* = 20
Complete relapse at 6 months after surgery	N/A: *n* = 11	N/A: *n* = 10	N/A: *n* = 21	—	—	N/A: *n* = 20
Complete relapse at 12 months after surgery	N/A: *n* = 11	N/A: *n* = 10	N/A: *n* = 10	—	—	N/A: *n* = 20
Patient's satisfaction	Yes: *n* = 11	N/A: *n* = 10	Yes: *n* = 11 N/A: *n* = 10	—	—	N/A: *n* = 20
Lip tension	Yes: *n* = 11	N/A: *n* = 10	Mild: *n* = 11 N/A: *n* = 10	—	—	N/A: *n* = 20
Pain	N/A: *n* = 11	N/A: *n* = 10	N/A: *n* = 21	—	—	N/A: *n* = 20
Perioral numbness	No: *n* = 11	N/A: *n* = 10	Yes: *n* = 3 No: *n* = 8 N/A: *n* = 10	—	—	N/A: *n* = 20
Quasiexperimental studies without control group: *N* = 2, *n* = 29	*N* = 0, *n* = 0	*N* = 0, *n* = 0	*N* = 0, *n* = 0	*N* = 0, *n* = 0	*N* = 0, *n* = 0	*N* = 2, *n* = 29
Mean GD change from baseline to 3rd month, mm	—	—	—	—	—	WMD: -3.92 95% CI: -4.47 to -3.36
Mean GD change from baseline to 6th month	—	—	—	—	—	WMD: -3.22 95% CI: -5.61 to -0.84
Mean GD change from baseline to 12th month	—	—	—	—	—	N/A (follow-up less than 12 months)
Success of treatment at 6 months after surgery	—	—	—	—	—	Yes = 11 No = 2 N/A: *n* = 16
Success of treatment at 12 months after surgery	—	—	—	—	—	N/A: *n* = 29
Stability at 6 months after surgery	—	—	—	—	—	N/A: *n* = 29
Stability at 12 months after surgery	—	—	—	—	—	N/A: *n* = 29
Complete relapse at 6 months after surgery	—	—	—	—	—	N/A: *n* = 29
Complete relapse at 12 months after surgery	—	—	—	—	—	N/A: *n* = 29
Patient's satisfaction	—	—	—	—	—	Yes: *n* = 12 No: *n* = 1 N/A: *n* = 16
Lip tension	—	—	—	—	—	Mild: *n* = 10 No: *n* = 3 N/A: *n* = 16
Pain	—	—	—	—	—	N/A: *n* = 29
Perioral numbness	—	—	—	—	—	Yes: *n* = 1 No: *n* = 28
Case series: *N* = 7, *n* = 43	*N* = 1, *n* = 12	*N* = 1, *n* = 1	*N* = 4, *n* = 28	*N* = 0, *n* = 0	*N* = 0, *n* = 0	*N* = 1, *n* = 2
Mean GD change from baseline to 3rd month, mm	*N* = 1, *n* = 12 MD: -1.92 95% CI: -2.53 to -1.31	N/A: *n* = 1	N/A: *n* = 28	—	—	N/A: *n* = 2
Mean GD change from baseline to 6th month	*N* = 1, *n* = 12 MD: -1.92 95% CI: -2.53 to -1.31	N/A: *n* = 1	*N* = 1, *n* = 10 MD: -3.60 95% CI: -4.66 to -2.54	—	—	N/A: *n* = 2
Mean GD change from baseline to 12th month	*N* = 1, *n* = 12 MD: -1.92 95% CI: -2.53 to -1.31	N/A: *n* = 1	*N* = 1, *n* = 10 MD: -3.50 95% CI: -4.60 to -2.40	—	—	N/A: *n* = 2
Success of treatment at 6 months after surgery	Yes: *n* = 8 No: *n* = 4	N/A: *n* = 1	N/A: *n* = 28	—	—	N/A: *n* = 2
Success of treatment at 12 months after surgery	Yes: *n* = 8 No: *n* = 4	N/A: *n* = 1	N/A: *n* = 28	—	—	N/A: *n* = 2
Stability at 6 months after surgery	Yes: *n* = 8 No: *n* = 4	N/A: *n* = 1	N/A: *n* = 28	—	—	N/A: *n* = 2
Stability at 12 months after surgery	Yes: *n* = 8 No: *n* = 4	N/A: *n* = 1	N/A: *n* = 28	—	—	N/A: *n* = 2
Complete relapse at 6 months after surgery	Yes: *n* = 1 No: *n* = 11	N/A: *n* = 1	N/A: *n* = 28	—	—	N/A: *n* = 2
Complete relapse at 12 months after surgery	Yes: *n* = 1 No: *n* = 11	N/A: *n* = 1	N/A: *n* = 28	—	—	N/A: *n* = 2
Patient's satisfaction	Yes: *n* = 8 No: *n* = 4	Yes: *n* = 1	Yes: *n* = 25 No: *n* = 3	—	—	Yes: *n* = 2
Lip tension	N/A: *n* = 12	N/A: *n* = 1	Slight: *n* = 1 No: *n* = 10 N/A: *n* = 17	—	—	Slight: *n* = 2
Pain	Mild: *n* = 12	N/A: *n* = 1	Mild: *n* = 11 N/A: *n* = 17	—	—	Mild: *n* = 2
Perioral numbness	No: *n* = 12	N/A: *n* = 1	No: *n* = 17 N/A: *n* = 11	—	—	N/A: *n* = 2
Case reports: *N* = 26, *n* = 26	*N* = 0, *n* = 0	*N* = 1, *n* = 1	*N* = 18, *n* = 18	*N* = 0, *n* = 0	*N* = 0, *n* = 0	*N* = 7, *n* = 7
Mean GD change from baseline to 3rd month, mm	—	N/A	*N* = 4, *n* = 4 MD = −4.62	—	—	*N* = 2, *n* = 2 MD: -3.25
Mean GD change from baseline to 6th month	—	N/A	*N* = 1, *n* = 1 MD: -6.75	—	—	*N* = 2, *n* = 2 MD: -6.50
Mean GD change from baseline to 12th month	—	N/A	*N* = 1, *n* = 1 MD: -6.00	—	—	N/A: *n* = 7
Success of treatment at 6 months after surgery	—	N/A: *n* = 1	Yes: *n* = 1 N/A: *n* = 17	—	—	Yes: *n* = 2 N/A: *n* = 5
Success of treatment at 12 months after surgery	—	N/A: *n* = 1	Yes: *n* = 1 N/A: *n* = 17	—	—	N/A: *n* = 7
Stability at 6 months after surgery	—	N/A: *n* = 1	No: *n* = 1 N/A: *n* = 17	—	—	No: *n* = 2 N/A: *n* = 5
Stability at 12 months after surgery	—	N/A: *n* = 1	No: *n* = 1 N/A: *n* = 17	—	—	N/A: *n* = 7
Complete relapse at 6 months after surgery	—	N/A: *n* = 1	No: *n* = 1 N/A: *n* = 17	—	—	No: *n* = 2 N/A: *n* = 5
Complete relapse at 12 months after surgery	—	N/A: *n* = 1	No: *n* = 1 N/A: *n* = 17	—	—	N/A: *n* = 7
Patient's satisfaction	—	Yes: *n*=1	Yes: *n* = 10 N/A: *n* = 8	—	—	Yes: *n* = 6 N/A: *n* = 1
Lip tension	—	N/A: *n* = 1	Slight: *n* = 6 Mild: *n* = 1 N/A: *n* = 11	—	—	Mild: *n* = 1 N/A: *n* = 6
Pain	—	N/A: *n* = 1	Mild: *n* = 9 N/A: *n* = 9	—	—	Mild: *n* = 2 N/A: *n* = 5
Perioral numbness	—	N/A: *n* = 1	Yes: *n* = 1 No: *n* = 1 N/A: *n* = 16	—	—	N/A: *n* = 7
All studies *N* = 38, *n* = 160	(*N* = 2, *n* = 23)	(*N* = 3, *n* = 12)	(*N* = 24, *n* = 67)	(*N* = 0, *n* = 0)	(*N* = 0, *n* = 0)	(*N* = 11, *n* = 58)
Mean GD change from baseline to 3rd month, mm	*N* = 2, *n* = 23 WMD: -2.98 95% CI: -5.10 to -0.85	*N* = 1, *n* = 10 MD: -3.29 95% CI: -4.70 to -1.88	*N* = 2, *n* = 21 WMD: -2.94 95% CI: -3.53 to -2.34	—	—	*N* = 3, *n* = 49 WMD: -3.71 95% CI: -3.99 to -3.42
Mean GD change from baseline to 6th month	*N* = 2, *n* = 23 WMD: -2.90 95% CI: -4.85 to -0.95	*N* = 1, *n* = 10 MD: -2.87 95% CI: -4.36 to -1.38	*N* = 3, *n* = 31 WMD: -2.68 95% CI: -3.49 to -1.86	—	—	*N* = 2, *n* = 29 WMD: -3.22 95% CI: -5.61 to -0.84
Mean GD change from baseline to 12th month	*N* = 1, *n* = 12 MD: -2.53 95% CI: -2.53 to -1.31	*N* = 1, *n* = 10 MD: -2.72 95% CI: -4.29 to -1.15	*N* = 2, *n* = 20 MD: -2.52 95% CI: -4.40 to -0.64	—	—	N/A
Success of treatment at 6 months after surgery	Yes: *n* = 8 No: *n* = 4 N/A: *n* = 11	N/A: *n* = 12	No: *n* = 1 N/A: *n* = 66	—	—	Yes: *n* = 13 No: *n* = 2 N/A: *n* = 43
Success of treatment at 12 months after surgery	Yes: *n* = 8 No: *n* = 4 N/A: *n* = 11	N/A: *n* = 12	No: *n* = 1 N/A: *n* = 55	—	—	N/A: *n* = 58
Stability at 6 months after surgery	Yes: *n* = 8 No: *n* = 4 N/A: *n* = 11	N/A: *n* = 12	No: *n* = 1 N/A: *n* = 66	—	—	No: *n* = 2 N/A: *n* = 56
Stability at 12 months after surgery	Yes: *n* = 8 No: *n* = 4 N/A: *n* = 11	N/A: *n* = 12	No: *n* = 1 N/A: *n* = 55	—	—	N/A: *n* = 58
Complete relapse at 6 months after surgery	Yes: *n* = 1 No: *n* = 11 N/A: *n* = 11	N/A: *n* = 12	No: *n* = 1 N/A: *n* = 66	—	—	No: *n* = 2 N/A: *n* = 56
Complete relapse at 12 months after surgery	Yes: *n* = 1 No: *n* = 11 N/A: *n* = 11	N/A: *n* = 12	No: *n* = 1 N/A: *n* = 55	—	—	N/A: *n* = 58
Patient's satisfaction	Yes: *n* = 19 No: *n* = 4	Yes: *n* = 2 N/A: *n* = 10	Yes: *n* = 46 No: *n* = 3 N/A: *n* = 18	—	—	Yes: *n* = 20 No: *n* = 1 N/A: *n* = 37
Lip tension	Yes: *n* = 11 N/A: *n* = 12	Yes: *n* = 0 N/A: *n* = 11	Slight: *n* = 7 Mild: *n* = 12 No: *n* = 10 N/A: *n* = 38	—	—	Slight: *n* = 2 Mild: *n* = 11 No: *n* = 3 N/A: *n* = 42
Pain	Mild: *n* = 12 N/A: *n* = 11	Yes: *n* = 1 N/A: *n* = 11	Mild: *n* = 20 N/A: *n* = 47	—	—	Mild: *n* = 4 N/A: *n* = 54
Perioral numbness	No: *n* = 23	Yes: *n* = 0 N/A: *n* = 11	Yes: *n* = 4 No: *n* = 26 N/A: *n* = 37	—	—	Yes: *n* = 1 No: *n* = 28 N/A: *n* = 29

Abbreviations: *N*: number of studies; *n*: number of patients; GD: gingival display; WMD: weighted mean difference; MD: mean difference; CI: confidence interval; GD: gingival display. ^∗^Clinical trials included one randomized clinical trial (RCT), one nonrandomized clinical trial, and one RCT with two arms of LRS and botulinum toxin type-A injection, in which we included the LRS arm in our study. Note: mean gingival display change = mean gingival display at endpoint–mean gingival display at baseline. ^#^Mean gingival display at month 4 minus mean gingival display at baseline. Note: stability of LRS surgery was considered only for studies with at least 6 months of follow-up. The result of LRS was considered stable if the amount of gingival display at 6 or 12 months was the same as that of obtained at 1 month. Complete relapse was considered only for studies with at least 6 months of follow-up. If the gingival display at 6 or 12 months was the same as that of baseline, we defined it as complete relapse at that time point. The result of LRS was considered a success if the amount of gingival display at 6 or 12 months was at most 3 mm at that time point.

## Supplementary Materials

Supplementary Materials Table S1: search syntax in PubMed database. Table S2: characteristics of excluded studies according to prespecified inclusion criteria and additional reasons. Table S3(a): summary of included clinical trials. Table S3(b): summary of included quasiexperimental studies. Table S3(c): summary of included case series. Table S3(d): summary of included case reports. Table S4(a): critical appraisal for quasiexperimental studies (nonrandomized experimental studies) included in the systematic review according to JBI. Table S4(b): critical appraisal for case-series studies included in the systematic review according to JBI. Table S4(c): critical appraisal for case reports included in the systematic review according to JBI.
