# Supplementary material for: Effectiveness of Different Modalities of Lip Repositioning Surgery for Management of Patients Complaining of Excessive Gingival Display: A Systematic Review and Meta-Analysis
Source: Biomed Res Int. 2021 Oct 7;2021:9476013. doi: 10.1155/2021/9476013 (PMC8516537; doi:10.1155/2021/9476013)
Supplement: Supplementary Materials — Table S1: search syntax in PubMed database. Table S2: characteristics of excluded studies according to prespecified inclusion criteria and additional reasons. Table S3(a): summary of included clinical trials. Table S3(b): summary of included quasiexperimental studies. Table S3(c): summary of included case series. Table S3(d): summary of included case reports. Table S4(a): critical appraisal for quasiexperimental studies (nonrandomized experimental studies) included in the systematic review according to JBI. Table S4(b): critical appraisal for case-series studies included in the systematic review according to JBI. Table S4(c): critical appraisal for case reports included in the systematic review according to JBI. [file 9476013.f1.docx]

| Table S1: Search syntax in PubMed database |
| --- |
| ((“gummy smile” OR (gummy AND smile) OR “excessive gingival show” OR (excessive AND “gingival show”) OR (excess AND “gingival show”) OR “excess gingival show” OR “excess gingival display” OR (excess AND “gingival display“) OR “excessive gingival display” OR (excessive AND “gingival display“) OR “hyperactive upper lip” OR (hyperactive AND “upper lip”) OR (“hyper-active” AND “upper lip”) OR “hyper-active upper lip” OR “hyper active upper lip” OR (“hyper active” AND “upper lip”) OR (hypermobile AND “upper lip”) OR (“hyper-mobile” AND “upper lip”) OR (“hyper mobile” AND “upper lip”) OR “high smile line” OR “high lip line” OR “vertical maxillary excess” OR “maxillary vertical excess” OR (“maxillary bone” AND excess) OR (“maxillary excess” AND bone)) AND (“lip stabilization” OR (lip AND stabilization) OR “lip reposition*” OR (lip AND reposition*) OR “lip re- position*” OR (lip AND re-position*) OR “vestibular shallowing” OR (position AND flap) OR myotomy OR “full thickness flap” OR (“full thickness” AND flap) OR “split thickness flap” OR (“split thickness” AND flap) “partial thickness flap” OR (“partial thickness” AND flap) OR frenectomy OR “frenum sparing” OR “frenulum sparing” OR “frenulum cut” OR (frenulum AND cut) OR (frenum AND cut) OR “tension free flap” OR “Tension-free flap” OR “flap advancement” OR (labial AND frenum AND cut))) OR ((“gummy smile” OR (gummy AND smile) OR “excessive gingival show” OR (excessive AND “gingival show”) OR (excess AND “gingival show”) OR “excess gingival show” OR “excess gingival display” OR (excess AND “gingival display“) OR “excessive gingival display” OR (excessive AND “gingival display“) OR “hyperactive upper lip” OR (hyperactive AND “upper lip”) OR (“hyper-active” AND “upper lip”) OR “hyper-active upper lip” OR “hyper active upper lip” OR (“hyper active” AND “upper lip”) OR (hypermobile AND “upper lip”) OR (“hyper-mobile” AND “upper lip”) OR (“hyper mobile” AND “upper lip”) OR “high smile line” OR “high lip line” OR “vertical maxillary excess” OR “maxillary vertical excess” OR (“maxillary bone” AND excess) OR (“maxillary excess” AND bone)) AND (“gingival show reduction” OR (“gingival show” AND reduction) OR (“gingival show” AND reduc*) OR (“gingival display” AND reduction) OR (“gingival display” AND reduc*) OR “gingival display reduction” OR (“postoperative” AND “gingival show”) OR (“post operative” AND “gingival show”) OR (post-operative AND “gingival show”) OR (“postoperative” AND “gingival display”) OR (“post-operative” AND “gingival display”) OR (post operative AND “gingival display”) OR relapse OR (lack AND stability) OR (“post operative” AND discomfort) OR (“post-operative” AND discomfort) OR (postoperative AND discomfort) OR “lip tension” OR (patient AND satisfaction) OR (patient AND satisfy*) OR (patient AND satisfied) OR (lip AND length) OR (“peri-oral” AND numbness) OR (perioral AND numbness) OR (“peri oral” AND numbness) OR (“post operative” AND pain) OR (“post-operative” AND pain) OR (postoperative AND pain))) |

| Table S2: Characteristics of excluded studies according to pre-specified inclusion criteria and additional reasons | | | | | | |
| --- | --- | --- | --- | --- | --- | --- |
| ID | Author’s name, year | Type of study | Human study | Over 18 years of old | Over 3 months follow up | Type of procedure |
| 1 | Nassif et al, N/M | Case report | Yes | Yes | No | LRS+CL |
| 2 | Benbrahim, N/M | Case report | Yes | Yes | N/M | LRS+Gingival contouring |
| 3 | Miskinyar, 1983 | Review | No (Review) | - | - | - |
| 4 | Robert A Levine, 1997 | Case report | Yes | Yes | Yes | CL+Gingivectomy |
| 5 | Ezquerra Carrera et al., 1999 | Case report | Yes | Yes | yes | LRS+Rhinoplasty+CL |
| 6 | Narayan, et al., 2011 | Case report | Yes | Yes | Yes | LRS+CL |
| 7 | Carinci et al., 2011 | Case report | Yes | Yes | Yes | LRS+Gingivectomy |
| 8 | Gupta et al., 2011 | Case report | Yes | Yes | No | LRS+Depigmentation+CL |
| 9 | Al-Dary, 2012 | Case report | Yes | Yes | N/M | LRS+CL |
| 10 | Espín et al., 2013 | Case report | Yes | Yes | No | CL+Implant surgury |
| 11 | Abou-Arraj et al., 2013 | Review | No (Review) | - | - | - |
| 12 | Dhruva et al., 2013 | Case report | Yes | Yes | No | LRS |
| 13 | Hegde et al., 2014 | Case report | Yes | Yes | No | Gingivectomy |
| 14 | Dayakar et al., 2014 | Case report | Yes | Yes | Yes | LRS+Undergoing orthodontic treatment |
| 15 | Jananni et al., 2014 | Case report | Yes | Yes | Yes | LRS+Undergoing orthodontic treatment |
| 16 | Balasubramaniam et al., 2014 | Case report | Yes | Yes | Yes | LRS+Undergoing orthodontic treatment |
| 17 | Narayanan et al., 2015 | Case report | Yes | Yes | N/M | LRS+Gingivectomy+Orthognathic surgery |
| 18 | Izraelewicz et al., 2015 | Review | No (Review) | - | - | - |
| 19 | Rao et al., 2015 | Case report | Yes | Yes | No | LRS |
| 20 | Manjunath et al., 2015 | Case report | Yes | Yes | Yes | LRS+Depigmentation+CL |
| 21 | Cimi Iqbal et al., 2015 | Case report | Yes | Yes | Yes | LRS+Depigmentation +Gingivoplasty |
| 22 | Hammouda et al., 2016 | Case series | Yes | Yes | No | LRS+Botox injection at 15^th^ day of LRS |
| 23 | Mahn , 2016 | Case report | Yes | Yes | No | LRS+CL |
| 24 | GEORGE et al., 2016 | Case report | Yes | Yes | N/M | LRS |
| 25 | Mantovani et al., 2016 | Case report | Yes | Yes | Yes | LRS+CL |
| 26 | Sharma et al., 2017 | Case report | Yes | Yes | No | LRS |
| 27 | Sánchez et al., 2017 | Case report | Yes | Yes | Yes | LRS+CL |
| 28 | Gonçalves et al., 2017 | Case report | Yes | Yes | Yes | CL |
| 29 | Bushra et al., 2017 | Case report | Yes | Yes | Yes | LRS+Undergoing orthodontic treatment |
| 30 | Makkiah, 2017 | RCT | Yes | N/M | Yes | LRS+One group Botox |
| 31 | Dannan, A et al., 2017 | RCT | Yes | Yes | No | LRS |
| 32 | Gibson et al., 2017 | Case report | Yes | No | Yes | LRS+CL |
| 33 | Farista et al., 2017 | Case report | Yes | Yes | Yes | LRS+Gingival contouring of right central |
| 34 | Krismariono., 2018 | Case report | Yes | Yes | No | LRS |
| 35 | Ahmed et al., 2018 | RCT | Yes | Yes | No | LRS+Intrusion of teeth |
| 36 | Humagain et al., 2018 | Review | No (Review) | - | - | - |
| 37 | Faus-Matoses et al., 2018 | Case report | Yes | Yes | Yes | LRS+Orthodontic treatment before LRS+CL |
| 38 | Deepthi et al., 2018 | Case report | Yes | Yes | No | LRS |
| 39 | Parihar, 2018 | Case report | Yes | Yes | No | LRS |
| 40 | Falcón-Guerrero, 2018 | Case report | Yes | Yes | No | LRS |
| 41 | Al-Hazmi, 2018 | Case report | Yes | Yes | No | LRS |
| 42 |  | Case report | Yes | Yes | No | LRS+Orthodontic treatment |
| 43 | Qassab et al., 2019 | RCT | Yes | N/M | No | LRS |
| 44 | Rameshet al., 2019 | Case series | Yes | Yes | Yes | LRS+CL: 2 case, undergoing orthodontics: 1 case) |
| 45 | Longo et al., 2019 | Case report | Yes | Yes | Yes | CL |
| 46 | Abhyankar., 2019 | Case report | Yes | Yes | Yes | LRS+CL |
| 47 | Aldelaimi et al., 2019 | RCT | Yes | Yes | No | LRS |
| 48 | Ganesh et al., 2019 | Case report | Yes | Yes | Yes | LRS+CL+Gingivectomy |
| 49 | Bilichodmath et al., 2019 | Case report | Yes | Yes | No | LRS |
| 50 | Bhimani et al., 2019 | Case report | Yes | Yes | Yes | LRS+Depigmentation+CL |
| 51 | Sharma Himani et al., 2019 | Case report | Yes | Yes | No | CL |
| 52 | Vij et al., 2020 | Case report | Yes | Yes | No | LRS+CL+Veneer |
| 53 | Boeire et al., 2020 | Case report | Yes | Yes | Yes | LRS+CL |
| 54 | Sangalette et al., 2020 | Case report | Yes | Yes | Yes | LRS+CL at one side |
| 55 | Prasant et al., 2020 | Case report | Yes | Yes | No | LRS |
| Excluded articles due to additional reasons during full text screening | | | | | | |
| 56 | Hamissi, 2015 | Case report | Inconsistency between surgical technique reported in text of the article and clinical photograph | | | |
| 57 | de Araújo et al., 2015 | Case report | Not reporting at least one of the outcomes of interest | | | |
| 58 | Trivedi et al., 2016 | Case report | Not reporting at least one of the outcomes of interest | | | |
| 59 | Kaushik et al., 2018 | Case report | Not reporting at least one of the outcomes of interest | | | |
| 60 | Alammar et al., 2018 | Prospective | Multiple publication | | | |
| 61 | Ashtaputre et al., 2012 | Case report | Unavailable full text* | | | |
| 62 | Montalvo-Arias et al., 2017 |  | Unavailable full text* | | | |
| Abbreviations: N/M, Not mentioned; CL, Crown Lengthening; LRS, Lip Repositioning Surgery; RCT, Randomized Controlled Trial | | | | | | |

| Table S3(a): Summary of included clinical trials | | | | | | | | | | | | | | | | | | | | | | | |
| --- | --- | --- | --- | --- | --- | --- | --- | --- | --- | --- | --- | --- | --- | --- | --- | --- | --- | --- | --- | --- | --- | --- | --- |
| **ID** | **Author, Year** | **Country** | **P  (No.)** | **Inclusion criteria** | **Intervention type** |  |  | |  | | | **Primary outcomes** | | | | | |  | **Secondary outcomes** | | | **follow-up time points** | **Comments  and complication** |
|  |  |  |  |  |  | **Pre-operative display (mm)** | **Post-operative display (mm)** | | | | | |  | **Stability** | **Complete relapse** | **Success of treatment** | **Patient’s satisfaction** |  | **Lip tension** | **Post-operative pain** | **Perioral numbness** |  |  |
|  |  |  |  |  |  |  | **1 m** | **3 m** | | **6 m** | **12 m** | |  |  |  |  |  |  |  |  |  |  |  |
| 1 | Alammar et al., 2018 | Syria | 11 | **Age:**  range: 18 to 38 years  **Gender:** 19 females and 3 males | **Modality 1:**  - Full-thickness  -Frenectomy  - First incision at 1 mm coronally to MGJ  - Distance between incisions: 10 to 12 mm  - Distal extension: mesial angle of first molar  - Myotomy | 6.36±1.12,  range: 5 to 8 | 0.91 ± 1.22 | 2.27 ± 1.27 | | 2.45 ± 1.13 | N/A | |  | At month 6: N/A: n=11  At month 12:  N/A: n=11 | At month 6: N/A: n=11  At month 12:  N/A: n=11 | At month 6: N/A: n=11  At month 12:  N/A: n=11 | 11 out of 11 were satisfied. |  | 11 out of 11 | N/A: n=11 | 3 out of 11  (First 3 weeks) | 1^st^ month,  3^rd^ month,  6^th^ month | - Blood oozing through suture in the initial days of the surgery  -Edema  -Flap dehiscence |
|  |  |  | 11 |  | **Modality 3:**  - Partial-thickness  - Frenectomy  - First incision at 1 mm coronally to MGJ  - Distance between incisions: 10 to 12 mm  - Distal extension: mesial angle of first molar | 5.82 ± 0.87  range: 5 to 7 | 2.18 ± 0.75 | 2.55 ± 0.93 | | 3.27 ± 0.79 | N/A | |  | At month 6: N/A: n=11  At month 12:  N/A: n=11 | At month 6: N/A: n=11  At month 12:  N/A: n=11 | At month 6: N/A: n=11  At month 12:  N/A: n=11 | 11 out of 11 were satisfied. |  | 11 out of 11 | N/A: n=11 | 0 out of 11 | 1^st^ month,  3^rd^ month,  6^th^ month |  |
| 2 | Tawfik et al., 2018 | Egypt | 10 | **Age:** N/A  range: N/A  **Gender:** 18 females and 2 males | **Modality 2:**  - Partial-thickness  - Frenectomy  - First incision at MGJ  - Distance between incisions: double to gingival display  - Distal extension: mesial angle of first molar  - Myotomy | 6.29±2.60 | N/A | 3.00±1.53 | | 3.42±1.23 | 3.57±1.62 | |  | At month 6: N/A: n=10  At month 12:  N/A: n=10 | At month 6: N/A: n=10  At month 12:  N/A: n=10 | At month 6: N/A: n=10  At month 12:  N/A: n=10 | N/A: n=10 |  | 0 out of 10 | N/A: n=10 | N/A: n=10 | 3rd month,  6th month,  12^th^ month | -Change in lip length (mm) with the lips in full rest separated into two measurements: vermillion length and philtrum length  -Post-operative swelling measured through facial contour changes  -Patient satisfaction (questionnaire)  -Post-operative pain visual analogue scale |
|  |  |  | 10 |  | **Modality 3:**  - Partial-thickness  - Frenectomy  - First incision at MGJ  - Distance between incisions: double to gingival display  - Distal extension: mesial angle of first molar | 4.31 ± 1.12 | N/A | 1.65± 0.90 | | 2.21 ± 1.00 | 2.73 ± 1.28 | |  | At month 6: N/A: n=10  At month 12:  N/A: n=10 | At month 6: N/A: n=10  At month 12:  N/A: n=10 | At month 6: N/A: n=10  At month 12:  N/A: n=10 | N/A: n=10 |  | 0 out of 10 | N/A: n=10 | N/A: n=10 | 3rd month,  6th month,  12^th^ month |  |
| 3 | Omer et al^*^., 2019 | Iraq | 20 | **Age**: 26.00 ± 5.20 years range: 18 to 35 years  **Gender**: All were females.  **Etiology:**  Short upper lip  Hypermobile upper lip | **Modality 6:**  - Partial thickness flap  - Without frenectomy  - First incision at MGJ  -Second incision with distance of 10 to 12 mm  -First molar | 4.88 ± 1.04 | 0.40 ± 0.55 | 1.31± 0.62 at month 4 | | N/A | N/A | |  | At month 6: N/A: n=20  At month 12:  N/A: n=20 | At month 6: N/A: n=20  At month 12:  N/A: n=20 | At month 6: N/A: n=20  At month 12:  N/A: n=20 | N/A: n=20 |  | 0 out of 20 | N/A: n=20 | N/A: n=20 |  |  |
| Abbreviations: MGJ, Mucogingival Junction; N/A: Not Available  # Additional data were provided via email.  Note: Stability of LRS surgery was considered only for studies with at least 6 months of follow-up. The result of LRS was considered as stable if the amount of gingival display at 6 or 12 months was the same as that of obtained at 1 month. Complete relapse was considered only for studies with at least 6 months of follow-up. If the gingival display at 6 or 12 months was the same as that of baseline, we defined it as complete relapse at that time point. The result of LRS was considered as success if the amount of gingival display at 6 or 12 months was at most 3 mm at that time point. | | | | | | | | | | | | | | | | | | | | | | | |

| Table S3 (b): Summary of included quasi-experimental studies | | | | | | | | | | | | | | | | | | | | | |
| --- | --- | --- | --- | --- | --- | --- | --- | --- | --- | --- | --- | --- | --- | --- | --- | --- | --- | --- | --- | --- | --- |
| **ID** | **Author, Year** | **Country** | **P  (No.)** | **Inclusion criteria** | **Intervention type** |  |  | | **Primary outcomes** | | | | | | |  | **Secondary outcomes** | | | **follow-up time points** | **Comments  and complication** |
|  |  |  |  |  |  | **Pre-operative display (mm)** | **Post-operative display (mm)** | | | | | **Stability** | **Complete relapse** | **Success of treatment** | **Patient’s satisfaction** |  | **Lip tension** | **Post-operative pain** | **Perioral numbness** |  |  |
|  |  |  |  |  |  |  | **1 m** | **3 m** | | **6 m** | **12 m** |  |  |  |  |  |  |  |  |  |  |
| 1 | Silva et al., 2013 | Brazil | 13 | **Age:** 28.7 ± 11.0 years, range: 19–49 years  **Gender:** 11 females and 2 males  **Etiology:** Hyperactive  upper lip | **Modality 6:**  - Partial-thickness  - Without frenectomy  - First incision at 1 mm coronally to MGJ  - Distance between incisions: 10 to 12 mm  - First molar | 5.8±2.1,  range: 4 to 10 | N/A | 1.4 ± 1.0 | | 1.3 ± 1.6 | N/A | At month 6: N/A: n=13  At month 12:  N/A: n=13 | At month 6: N/A: n=13  At month 12:  N/A: n=13 | At month 6: 11 out of 13  At month 12:  N/A: n=13 | 12 out of 13 patients were somewhat to extremely satisfied. |  | 10 out of 13 had mild lip tension (First week)  ----  After 2.5 Years, two out of 13 patients had very rarely lip tension | N/A | 1 out of 13 (First week) | 4^th^ week  3^rd^ month  6^th^ month | -A minor scar formation  -Upper lip length increased  -Upper lip vermillion length increase |
| 2 | Serhat İzol et al., 2019 | Turkey | 16 | **Age**: 29.35 ± 3.06 years range: 26 to 36 years  **Gender**: All were females.  **Etiology:** N/A | **Modality 6:**  - Partial-thickness  - Without frenectomy  - First incision at MGJ  - Distance between incisions: 10 to 12 mm  - First premolar | 4.93 ± 0.85 | N/A | 1.06± 0.98 | | 2.87± 0.8 | N/A | At month 6: N/A: n=16  At month 12:  N/A: n=16 | At month 6: N/A: n=16  At month 12:  N/A: n=16 | At month 6: N/A: n=16  At month 12:  N/A: n=16 | N/A |  | N/A | N/A | 0 out of 16^#^ | 3^rd^ month  6^th^ month | - Relapse between the 3^rd^ and 6^th^ month period following the operation was 1.68±0.60 mm |
| Abbreviations: MGJ, Mucogingival Junction; N/A: Not Available  # Additional data were provided via email.  Note: Stability of LRS surgery was considered only for studies with at least 6 months of follow-up. The result of LRS was considered as stable if the amount of gingival display at 6 or 12 months was the same as that of obtained at 1 month. Complete relapse was considered only for studies with at least 6 months of follow-up. If the gingival display at 6 or 12 months was the same as that of baseline, we defined it as complete relapse at that time point. The result of LRS was considered as success if the amount of gingival display at 6 or 12 months was at most 3 mm at that time point. | | | | | | | | | | | | | | | | | | | | | |

| Table S3 (c): Summary of included case series | | | | | | | | | | | | | | | | | | | | | |
| --- | --- | --- | --- | --- | --- | --- | --- | --- | --- | --- | --- | --- | --- | --- | --- | --- | --- | --- | --- | --- | --- |
| **ID** | **Author, Year** | **Country** | **P  (No.)** | **Inclusion criteria** | **Intervention type** |  |  | | **Primary outcomes** | | | | | | |  | **Secondary outcomes** | | | **follow-up time points** | **Comments  and complication** |
|  |  |  |  |  |  | **Pre-operative display (mm)** | **Post-operative display (mm)** | | | | | **Stability** | **Complete relapse** | **Success of treatment** | **Patient’s satisfaction** |  | **Lip tension** | **Post-operative pain** | **Perioral numbness** |  |  |
|  |  |  |  |  |  |  | **1 m** | **3 m** | | **6 m** | **12 m** |  |  |  |  |  |  |  |  |  |  |
| 1 | Abdullah et al., 2014 | 12 | Saudi Arabia | **Age:** 23.75±2.89 years  range: 20 to 29 years  **Gender:** 10 Females and 2 males  **Etiology:** N/A | **Modality 1:**  - full-thickness  - Frenectomy  -First incision at 4 to 5 mm above gingival margin  - Distance between incisions: 8 to 10 mm  - Distal extension: second premolar  - Myotomy | 5.00±0.95, | 2.66±0.77 | 3.08±1.16 | | 3.08±1.16 | 3.08±1.16 | At month 6: Yes: n=8, No: n=4  At month 12:  Yes: n=8, No: n=4 | At month 6: Yes: n=1, No: n=11  At month 12:  Yes: n=1, No: n=11 | At month 6: Yes: n=8, No: n=4  At month 12:  Yes: n=8, No: n=4 | 8 out of 12 were satisfied. |  | N/A: n=12 | All the patients had mild pain. | 0 out of 12^#^ | 1^st^ month,  3^rd^ month,  6^th^ month,  12^th^ month | -Edema in the perioral area that was extended to the lower eye lids with ecchymosis in one female patient which lasted for 2 weeks |
| 2 | Jacobs et al., 2013 | 7 | USA | **Age**: 21 to 59 years  **Gende**r: All were females.  **Etiology:** N/A | **Modality 3:**  - Reversible trial  - Partial- thickness  - Frenectomy  - First incision at MGJ  - Distance between incisions: twice the length of repositioning desired  - Distal extension: first molar  **Instrument used:**  Diode laser,  scalpel | 5.3±1.5 | N/A | N/A | | N/A | N/A | At month 6: N/A: n=7  At month 12:  N/A: n=7 | At month 6: N/A: n=7  At month 12:  N/A: n=7 | At month 6: N/A: n=7  At month 12:  N/A: n=7 | 6 out of 7 were satisfied. |  | N/A: n=7 | N/A: n=7 | 0 out of 7 | 1st month,  3^rd^ year | - |
| 3 | Ozturan et al., 2014 | 10 | Turkey | **Age**: 27.8 ± 9.0 years  range: 22 to 34 years  **Gender**: All were females.  **Etiology**: Hypermobile upper lip | **Modality 3:**  - Partial- thickness  - Frenectomy  - First incision at MGJ  - Distance between incisions:10 to12 mm  - Distal extension: first molar  **Instrument used:**  Diode laser | 4.73±1.83 | N/A | N/A | | 1.13±1.0 | 1.23±1.1 | At month 6: N/A: n=10  At month 12:  N/A: n=10 | At month 6: N/A: n=10  At month 12:  N/A: n=10 | At month 6: N/A: n=10  At month 12:  N/A: n=10 | All patients were satisfied. |  | N/A: n=10 | Mild: n=10 | N/A: n=10 | 6^th^ month,  1^st^ year | - |
| 4* | Khan.M et al.,2017 | 3 | India | **Age**: 18 year  **Gender**: females  **Etiology**: VME | **Modality 3:**  - Partial- thickness  - Frenectomy  - First incision at MGJ  - Distance between incisions:12 mm  - Distal extension: first molar | 6 | N/A | N/A | | N/A | N/A | At month 6: N/A: n=1  At month 12:  N/A: n=1 | At month 6: N/A: n=1  At month 12:  N/A: n=1 | At month 6: N/A: n=1  At month 12:  N/A: n=1 | One out of one was satisfied |  | Slight: n=1  (First week) | Mild: n=1 | N/A: n=1 | 6^th^ month | - |
| 5** | Torabi et al.,2018 | 3 | USA | **Age**: range: 41 to 54 years  **Gender**: 1 female and 1 male  **Etiology**: Case 1:VME and Hyperactive upper lip in  Case 2: N/A | **Modality 6:**  - Partial- thickness  - Without frenectomy  - First incision at MGJ  - Distance between incisions: twice the gingival display  - Distal extension: first molar | N/A^€^ | N/A^€^ | N/A^€^ | | N/A^€^ | N/A^€^ | At month 6: N/A: n=2  At month 12:  N/A: n=2 | At month 6: N/A: n=2  At month 12:  N/A: n=2 | At month 6: N/A: n=2  At month 12:  N/A: n=2 | All patients were satisfied. |  | Slight: n=2  (two to three weeks) | Mild: n=2  (First three days) | N/A: n=2 | 1^st^ week  2^nd^ week  3^rd^ week  2^nd^ month  6^th^ month  13^th^ month  16^th^ month | Minor scar formation |
| 6 | Ala’Ersheidat et al., 2019 | 10 | Jordan | **Age**: 22 to 28 years  **Gender**: All were females.  **Etiology**: N/A | **Modality 3:**  - Partial- thickness  - Frenectomy  - First incision at MGJ  - Distance between incisions: 12 mm  - Distal extension: Second pre molars | 6 to 8 | N/A | N/A | | N/A | N/A | At month 6: N/A: n=10  At month 12:  N/A: n=10 | At month 6: N/A: n=10  At month 12:  N/A: n=10 | At month 6: N/A: n=10  At month 12:  N/A: n=10 | 8 out of 10 were satisfied. |  | 0 out of 10^#^ | N/A: n=10 | 0 out of 10^#^ | 6^th^ month  12^th^ month  18^th^ month | At 18 months three patients were lost fir follow up and 4 were unsatisfied |
| 7*** | Zardawi et al.,2020 | 4 | Iraq | **Age**: 23 years  **Gender**: female  **Etiology**: N/A | **Modality 2:**  - Partial- thickness  - Frenectomy  - First incision at MGJ  - Distance between incisions: 5 mm  - Distal extension: Second pre molars  - Myotomy | 6 | N/A | N/A | | N/A | N/A | At month 6: N/A: n=1  At month 12:  N/A: n=1 | At month 6: N/A: n=1  At month 12:  N/A: n=1 | At month 6: N/A: n=1  At month 12:  N/A: n=1 | One out of one was satisfied |  | N/A: n=1 | N/A: n=1 | N/A: n=1 | 6^th^ month,  1^st^ year | - |
| Abbreviations: MGJ, Mucogingival Junction; VME: Vertical Maxillary Excess; N/A: Not Available  # Additional data were provided via email.  * The second patient was excluded due to not reporting at least one of the outcomes and the third patient was excluded due to orthodontics treatment.  ** One of the patients was excluded due to gingival countering  *** Only the first case of this article was eligible to be included.  € Two out of 3 patients of this case series were included in the current systematic review. Data for patients were not reported individually. Thus, it was impossible to calculate the mean gingival display for these two patients.  Note: Stability of LRS surgery was considered only for studies with at least 6 months of follow-up. The result of LRS was considered as stable if the amount of gingival display at 6 or 12 months was the same as that of obtained at 1 month. Complete relapse was considered only for studies with at least 6 months of follow-up. If the gingival display at 6 or 12 months was the same as that of baseline, we defined it as complete relapse at that time point. The result of LRS was considered as success if the amount of gingival display at 6 or 12 months was at most 3 mm at that time point. | | | | | | | | | | | | | | | | | | | | | |

| Table S3 (d): Summary of included case reports | | | | | | | | | | | | | | | | | | | | | |
| --- | --- | --- | --- | --- | --- | --- | --- | --- | --- | --- | --- | --- | --- | --- | --- | --- | --- | --- | --- | --- | --- |
| **ID** | **Author, Year** | **Country** | **P  (No.)** | **Inclusion criteria** | **Intervention type** |  |  | | **Primary outcomes** | | | | | | |  | **Secondary outcomes** | | | **follow-up time points** | **Comments  and complications** |
|  |  |  |  |  |  | **Pre-operative display (mm)** | **Post-operative display (mm)** | | | | | **Stability** | **Complete relapse** | **Success of treatment** | **Patient’s satisfaction** |  | **Lip tension** | **Post-operative pain** | **Perioral numbness** |  |  |
|  |  |  |  |  |  |  | **1 m** | **3 m** | | **6 m** | **12 m** |  |  |  |  |  |  |  |  |  |  |
| 1 | Rosenblatt et al., 2006 | USA | 1 | **Age:** 30  **Gender:**  Female  **Etiology:** N/A | **Modality 3:**  - Partial-thickness  - Frenectomy  - First incision at MGJ  - Distance between incisions: 10 to 12 mm  - Distal extension: mesial of first molar | 3 to 4 | N/A | N/A | | N/A | N/A | At month 6: N/A  At month 12: N/A | At month 6: N/A  At month 12: N/A | At month 6: N/A  At month 12: N/A | N/A |  | Slight  (Fist week) | Mild | N/A | 2^nd^ week,  8^th^ month | - Scar formation  - Gingival display reduction |
| 2 | Simon et al., 2007 | USA | 1 | **Age:** 34  **Gender:**  Female  **Etiology:** Moderate VME | **Modality 3:**  - Partial-thickness  - Frenectomy  - First incision at MGJ  - Distance between incisions: 10 to 12 mm  - Distal extension: first molar | N/A | N/A | N/A | | N/A | N/A | At month 6: N/A  At month 12: N/A | At month 6: N/A  At month 12: N/A | At month 6: N/A  At month 12: N/A | N/A |  | N/A | Mild  (First two days) | N/A | 3^rd^ Month,  1^st^ Year | - Mild swelling  - Upper lip tension  - Gingival display reduction |
| 3 | Gupta et al., 2010 | India | 1 | **Age:** 25  **Gender:**  Female  **Etiology:** Incompetent  lips | **Modality 3:**  - Partial-thickness  - Frenectomy  - First incision at MGJ  - Distance between incisions: 10 to 12 mm  - Distal extension: mesial of first molar | N/A | N/A | N/A | | N/A | N/A | At month 6: N/A  At month 12: N/A | At month 6: N/A  At month 12: N/A | At month 6: N/A  At month 12: N/A | N/A |  | Mild  (First week) | Mild  (First week) | N/A | 1^ST^ week,  3^rd^ month,  6^th^ month | - Scar formation |
| 4 | Humayun et al., 2010 | USA | 1 | **Age:** 30  **Gender:** Female  **Etiology:** Degree I VME, Hypermobility, Local altered  passive  eruption | **Modality 3:**  - Partial-thickness  - Frenectomy  - First incision at MGJ  - Distance between incisions: 14 to 16 mm  - Distal extension: second premolar | 2 to 4 | N/A | N/A | | N/A | N/A | At month 6: N/A  At month 12: N/A | At month 6: N/A  At month 12: N/A | At month 6: N/A  At month 12: N/A | Satisfied |  | N/A | N/A | N/A | 1^st^ week,  2^nd^ week,  4^th^ week,  11^th^ month,  1^st^ year | - Scar formation  - Extra oral swelling  - Bruising  - Slight erythema |
| 5 | Sheth et al., 2013 | India | 1 | **Age:** 35  **Gender:** Females  **Etiology:** Gingival  disharmony | **Modality 3:**  - Partial-thickness  - Frenectomy  - First incision at MGJ  - Distance between incisions: 10 to 12 mm  - Distal extension: mesial of first premolar | 8 to 10 | N/A | 4 to 6 | | N/A | N/A | At month 6: N/A  At month 12: N/A | At month 6: N/A  At month 12: N/A | At month 6: N/A  At month 12: N/A | Satisfied |  | Slight | Mild | N/A | N/A | - Scar formation  - Mild swelling |
| 6* | Vital Ribeiro-Júnior et al., 2013 | Brazil | 2 | **Age**: 21  **Gender**: Female  **Etiology**: Hypermobility of upper lip | **Modality 6:**  - Partial-thickness  - Without frenectomy  - First incision at 1 mm coronally to MGJ  - Distance between incisions: 10 to 12 mm  - Distal extension: first molar | 7 | N/A | N/A | | 1 | N/A | At month 6: N/A  At month 12: N/A | At month 6: No  At month 12: N/A | At month 6: Yes  At month 12: N/A | Satisfied |  | N/A | N/A | N/A | 6^th^ month | - Scar formation |
| 7 | Gaddale et al., 2014 | India | 1 | **Age**: 25  **Gender**: Female  **Etiology**: Moderate VME, Hypermobility of upper lip | **Modality 3:**  - Partial-thickness  - Frenectomy  - First incision at MGJ  - Distance between incisions: 13 to 15 mm  - Distal extension: first premolar | 7^#^ | N/A | N/A | | N/A | N/A | At month 6: N/A  At month 12: N/A | At month 6: N/A  At month 12: N/A | At month 6: N/A  At month 12: N/A | Satisfied^#^ |  | Slight^#^ | Mild | 0 out of 1^#^ | 1^st^ week,  2^nd^ month,  1^st^ year | - Scar formation  - Gingival display reduction |
| 8 | Grover et al.,2014 | India | 1 | **Age**: 18  **Gender**: Female  **Etiology**: Short lip, Incompetent lips | **Modality 3:**  - Partial-thickness  - Frenectomy  - First incision at MGJ  - Distance between incisions: 8 to 10 mm  - Distal extension: first molar  - Flap advancement | 4 to 5 | N/A | N/A | | N/A | N/A | At month 6: N/A  At month 12: N/A | At month 6: N/A  At month 12: N/A | At month 6: N/A  At month 12: N/A | N/A |  | N/A | Mild  (First week) | N/A | 2^nd^ week,  1^st^ year | - Scar formation |
| 9 | Pandurić et al., 2014 | Republic of Croatia | 1 | **Age**: 27  **Gender**: Female  **Etiology**: Hyperactive upper lip, Altered passive eruption, VME | **Modality 3:**  - Reversible trial  - Partial-thickness  - Frenectomy  - First incision at MGJ  - Distance between incisions: 7 to 12 mm  - Distal extension: first molar  - Instrument: scalpel and diode laser | 5.5 to 10 | N/A | 0 to 2 | | 0 to 2 | N/A | At month 6: N/A  At month 12: N/A | At month 6: No  At month 12: N/A | At month 6: Yes  At month 12: N/A | Satisfied |  | Slight  (First week) | Mild  (First week) | 1 out of 1  (First week) | 10^th^ day,  14^th^ day,  3^rd^ month,  6^th^ month | - Scar formation  - Upper lip length increased  - Upper lip vermillion length |
| 10 | Muthukumar et al., 2015 | India | 1 | **Age**: 32  **Gender**: Female  **Etiology**: Incompetent lips | **Modality 3:**  - Partial-thickness  - Frenectomy  - First incision at MGJ  - Distance between incisions: 10 to 12 mm  - Distal extension: second premolar | 5 to 6 | N/A | N/A | | N/A | N/A | At month 6: N/A  At month 12: N/A | At month 6: N/A  At month 12: N/A | At month 6: N/A  At month 12: N/A | N/A |  | N/A | N/A | N/A | 1^st^ week,  2^nd^ week,  1^st^ year | - Minimal postoperative complications for one week |
| 11 | Sthapak et al., 2015 | India | 1 | **Age**: 25  **Gender**: Female  **Etiology**: Incompetent lips | **Modality 6:**  - Partial-thickness  - Without frenectomy  - First incision at 1 mm coronally to MGJ  - Distance between incisions: 10 to 12 mm  - Distal extension: premolar | 7 | N/A | 3 | | N/A | N/A | At month 6: N/A  At month 12: N/A | At month 6: N/A  At month 12: N/A | At month 6: N/A  At month 12: N/A | Satisfied |  | N/A | N/A | N/A | 1^st^ week,  1^st^ month,  3^rd^ month | - Minor scar formation |
| 12 | Tasdemir et al., 2015 | Turkey | 1 | **Age**: 22  **Gender**: Female  **Etiology**: Delayed tooth eruption, Hyperactive upper | **Modality 6:**  - Partial-thickness  - Without frenectomy  - First incision at 1 mm coronally to MGJ  - Distance between incisions: 10 to 12 mm  - Distal extension: first molar | N/A | N/A | N/A | | N/A | N/A | At month 6: N/A  At month 12: N/A | At month 6: N/A  At month 12: N/A | At month 6: N/A  At month 12: N/A | Satisfied |  | N/A | N/A | N/A | 1^st^ year | - Tension for 1 week  - Minor scar formation  - Gingival display reduction |
| 13 | Pawar et al., 2015 | India | 1 | **Age**: 19  **Gender**: Female  **Etiology**: Moderate VME | **Modality 3:**  - Partial-thickness  - Frenectomy  - First incision at MGJ  - Distance between incisions: 10 to 12 mm  - Distal extension: second premolar | N/A | N/A | N/A | | N/A | N/A | At month 6: N/A  At month 12: N/A | At month 6: N/A  At month 12: N/A | At month 6: N/A  At month 12: N/A | Satisfied |  | N/A | N/A | N/A | 1^st^ week,  1^st^ month,  6^th^ month | N/A |
| 14 | Funde et al., 2016 | India | 1 | **Age**: 21  **Gender**: Female  **Etiology**: Class II malocclusion, VME | **Modality 3:**  - Reversible trial  - Partial-thickness  - Frenectomy  - First incision at MGJ  - Distance between incisions: N/A  - Distal extension: molar | 5 to 7 | 1.5 to 2 | 3.5 to 5 | | N/A | N/A | At month 6: N/A  At month 12: N/A | At month 6: N/A  At month 12: N/A | At month 6: N/A  At month 12: N/A | Satisfied |  | N/A | Mild  (the day after surgery) | N/A | 3^rd^ month | N/A |
| 15 | Littuma, et al., 2017 | Brazil | 1 | **Age**: 21  **Gender**: Male  **Etiology**: Hyperactive upper lip | **Modality 6:**  - Partial-thickness  - Without frenectomy  - First incision at 1 mm coronally to MGJ  - Distance between incisions: 8 to 10 mm  - Distal extension: first molar | 4 to 5 | N/A | N/A | | N/A | N/A | At month 6: N/A  At month 12: N/A | At month 6: N/A  At month 12: N/A | At month 6: N/A  At month 12: N/A | N/A |  | Mild  (First week) | Mild  (First week) | N/A | 1^st^ week,  1^st^ year | - Minor scar formation  - Gingival display reduction |
| 16 | Moideen et al., 2017 | India | 1 | **Age**: 38  **Gender:** Female  **Etiology**: VME | **Modality 6:**  - Partial-thickness  - Without frenectomy  - First incision at MGJ  - Distance between incisions: 10 mm  - Distal extension: mesial of first premolar | 5 | N/A | 2 to 3 | | N/A | N/A | At month 6: N/A  At month 12: N/A | At month 6: N/A  At month 12: N/A | At month 6: N/A  At month 12: N/A | Satisfied |  | N/A | N/A | N/A | 3^rd^ month | - Reduction in probing depth  - tension for one week |
| 17 | Ambrosio et al., 2018 | USA | 2 | **Age**: Both 27  **Gender**: both female  **Etiology**: Severe VME, Hypermobile short upper lip, Incompetent lip | **Modality 3:**  - Partial-thickness  - Frenectomy  - First incision at MGJ  - Distance between incisions: 12 mm  - Distal extension: second premolar | 11 | N/A | N/A | | N/A | N/A | At month 6: N/A  At month 12: N/A | At month 6: N/A  At month 12: N/A | At month 6: N/A  At month 12: N/A | Satisfied |  | N/A | N/A | N/A | 1^st^ month,  1^st^ year,  2^nd^ year | - Bilateral swelling and ecchymosis in the peri-oral and peri-orbital areas |
| 18 | Mathew ,2018 | India | 1 | **Age**: 26  **Gender**: Male  **Etiology:**  VME Hyperactive upper lip | **Modality 6:**  - Partial-thickness  - Without frenectomy  - First incision at 1 mm coronally to MGJ  - Distance between incisions: 16 mm  - Distal extension: first molar | N/A | N/A | N/A | | N/A | N/A | At month 6: N/A  At month 12: N/A | At month 6: N/A  At month 12: N/A | At month 6: N/A  At month 12: N/A | Satisfied |  | N/A | N/A | N/A | 2^nd^ week,  6^th^ month | - Minimal discomfort  - Minor scar formation |
| 19* | Gwi-Hyeon Min et al*.,2018 | Korea | 3 | **Age**: 35  **Gender**: female  **Etiology:** Hyperactive upper lip | **Modality 3:**  - Partial-thickness  - Frenectomy  - First incision at MGJ  - Distance between incisions: 12 mm  - Distal extension: second premolar | 8 | N/A | 2 | | N/A | 2 | At month 6: N/A  At month 12: N/A | At month 6: N/A  At month 12: No | At month 6: N/A  At month 12: yes | N/A |  | N/A | N/A | N/A | 6^th^ month,  1^st^ year | -at 3 month of post-operative tendency to recur |
| 20 | Foudah, 2019 | Saudi Arabia | 1 | **Age**: 25  **Gender**: Female  **Etiology:** VME II, Hypermobile upper lip | **Modality 3:**  - Partial-thickness  - Frenectomy  - First incision at MGJ  - Distance between incisions: 10 to 14 mm  - Distal extension: first molar | 5 to7 ^#^ | N/A | N/A | | N/A | N/A | At month 6: N/A  At month 12: N/A | At month 6: N/A  At month 12: N/A | At month 6: N/A  At month 12: N/A | N/A |  | N/A | N/A | 0 out of 1^#^ | 1^st^ week,  2^nd^ week,  4^th^ year | -Little swelling and restriction in the lip movement at 1^st^ week  - Scar formation |
| 21 | Gadalla et al.,2019 | USA | 1 | **Age**: 26  **Gender**: Female  **Etiology:** VME class two, Hypermobile upper lip, Incompetent lip, Mouth breathing | **Modality 3:**  - Partial-thickness  - Frenectomy  - First incision at MGJ  - Distance between incisions: 10 mm  - Distal extension: first premolar  - Flap advancement | 8 | N/A | N/A | | N/A | N/A | At month 6: N/A  At month 12: N/A | At month 6: N/A  At month 12: N/A | At month 6: N/A  At month 12: N/A | Satisfied |  | N/A | N/A | N/A | 2^nd^ week,  4^th^ week,  6^th^ week,  6^th^ month,  12^th^ month | - No post op complications were noticed |
| 22 | Mohanty et ,al. 2019 | India | 1 | **Age**: 20 years  **Gender**: Female  **Etiology:** Severe VME | **Modality 3:**  - Partial-thickness  - Frenectomy  - First incision at MGJ  - Distance between incisions: 11 to 12 mm  - Distal extension: first premolar | 5 to 6 | N/A | N/A | | N/A | N/A | At month 6: N/A  At month 12: N/A | At month 6: N/A  At month 12: N/A | At month 6: N/A  At month 12: N/A | Satisfied |  | slight^#^ | Mild^#^ | N/A | 1^st^ month,  3^rd^ month | - Gingival display reduction  - Tension around the upper lip without any swelling for one week |
| 23 | Thaker et al.,2019 | India | 1 | **Age**: 37  **Gender**: Female  **Etiology:** Hyperactive upper lip | **Modality 3:**  - Partial-thickness  - Frenectomy  - First incision at MGJ  - Distance between incisions: 10 to 12 mm  - Distal extension: mesial of premolar  -Flap advancement | 10 | N/A | N/A | | N/A | N/A | At month 6: N/A  At month 12: N/A | At month 6: N/A  At month 12: N/A | At month 6: N/A  At month 12: N/A | Satisfied |  | Slight  (First week) | N/A | N/A | 2^nd^ week,  6^th^ month | - Minimal bruising,  - Minimal extra oral swelling  - Scar formation  - The gingival display measured after 1 week it was <1 mm |
| 24 | Iikura et al. 2019 | Tokyo | 1 | **Age**: 24  **Gender**: Female  **Etiology:**  Hyperactive upper lip | **Modality 6:**  - Reversible trial  - Partial-thickness  - Without frenectomy  - First incision at MGJ  - Distance between incisions: N/A  - Distal extension: first molar | 8 | N/A | N/A | | 1 | N/A | At month 6: N/A  At month 12: N/A | At month 6: No  At month 12: N/A | At month 6: yes  At month 12: N/A | Satisfied |  | N/A | Mild | N/A | 1^st^ month,  3^rd^ month,  6^th^ month,  1^st^ year | - After one week swelling and bleeding  - Difficulty of speaking |
| 25 | Duruel et al., 2020 | USA | 1 | **Age**: 24  **Gender**: Female  **Etiology:** Hyperactivity of muscles | **Modality 2:**  - Partial-thickness  - Frenectomy  - First incision at MGJ  - Distance between incisions: twice gingival display  - Distal extension: second premolar  - Myotomy | 4.7 | 2.38 | N/A | | N/A | N/A | At month 6: N/A  At month 12: N/A | At month 6: N/A  At month 12: N/A | At month 6: N/A  At month 12: N/A | Satisfied |  | N/A | N/A | N/A | 10^th^ day,  1^st^ month,  3^rd^ month | N/A |
| 26 | Sharma et al.,2020 | India | 1 | **Age**: 23  **Gender**: Female  **Etiology**: Maxillary anterior retraction | **Modality 3:**  - Partial-thickness  - Frenectomy  - First incision at MGJ  - Distance between incisions: 10 mm  - Distal extension: first molar | 8 to 10 | N/A | N/A | | N/A | N/A | At month 6: N/A  At month 12: N/A | At month 6: N/A  At month 12: N/A | At month 6: N/A  At month 12: N/A | N/A. |  | N/A | N/A | N/A | 2^nd^ week,  6^th^ month | - No complication was recorded  - Reduction of 4-5mm was noticed with 3mm gingival display remaining as measured 2 weeks after the surgery |
| Abbreviations: MGJ: Mucogingival Junction; VME: Vertical Maxillary Excess; N/A: Not Available  *Just first case included  # Additional data were provided via email.  Note: Stability of LRS surgery was considered only for studies with at least 6 months of follow-up. The result of LRS was considered as stable if the amount of gingival display at 6 or 12 months was the same as that of obtained at 1 month. Complete relapse was considered only for studies with at least 6 months of follow-up. If the gingival display at 6 or 12 months was the same as that of baseline, we defined it as complete relapse at that time point. The result of LRS was considered as success if the amount of gingival display at 6 or 12 months was at most 3 mm at that time point. | | | | | | | | | | | | | | | | | | | | | |

| Table S4 (a). Critical appraisal for Quasi-Experimental Studies (non-randomized experimental studies) included in the systematic review according to JBI | | | | | | | | | | | |
| --- | --- | --- | --- | --- | --- | --- | --- | --- | --- | --- | --- |
| ID | Author, year | Q1 | Q2 | Q3 | Q4 | Q5 | Q6 | Q7 | Q8 | Q9 | Total score^*^ |
| 1 | Silva et al., 2013 | Yes | Yes | Yes | No | Yes | Yes | Yes | Yes | No | 7 |
| 2 | Serhat İzol et al., 2019 | Yes | Yes | Yes | No | Yes | Yes | Yes | Unclear | No | 6 |
| 1. Abbreviations: JBI, Joanna Briggs Institute 2. Note: Q1 to Q9 indicate questions 1 to 9 based on the JBI critical appraisal checklist as following: 3. Q1- Is it clear in the study what is the ‘cause’ and what is the ‘effect’ (i.e. there is no confusion about which variable comes first)? 4. Q2- Were the participants included in any comparisons similar? 5. Q3- Were the participants included in any comparisons receiving similar treatment/care, other than the exposure or intervention of interest? 6. Q4- Was there a control group? 7. Q5- Were there multiple measurements of the outcome both pre and post the intervention/exposure? 8. Q6- Was follow up complete and if not, were differences between groups in terms of their follow up adequately described and analyzed? 9. Q7- Were the outcomes of participants included in any comparisons measured in the same way? 10. Q8- Were outcomes measured in a reliable way? 11. Q9-Was appropriate statistical analysis used? 12. * The JBI checklist has following four responses: Yes, No, Unclear, Not applicable. For "Yes" will be equal to 1 point, and 0 point will be considered for other responses. 13. ^#^ Omer et al. study is a randomized clinical trial with two arms including modified lip repositioning surgery (MLRS) and non-surgical technique using BTX-A injection, Botox (Allergan). According to our inclusion criteria, only the MLRS group was included in our study. | | | | | | | | | | | |

| Table S4 (b). Critical appraisal for case-series studies included in the systematic review according to JBI | | | | | | | | | | | | |
| --- | --- | --- | --- | --- | --- | --- | --- | --- | --- | --- | --- | --- |
| ID | Author, year | Q1 | Q2 | Q3 | Q4 | Q5 | Q6 | Q7 | Q8 | Q9 | Q10 | Total score* |
| 1 | Jacobs et al., 2013 | No | No | No | Yes | Yes | Unclear | No | Yes | No | Not applicable | 3 |
| 2 | Abdullah et al., 2014 | Yes | Yes | Yes | No | Yes | Yes | Unclear | Yes | No | Yes | 7 |
| 3 | Ozturan et al., 2014 | Yes | Yes | Yes | No | Yes | Yes | Unclear | Yes | No | Yes | 7 |
| 4 | Khan.M et al., 2017 | No | Yes | Yes | No | Yes | Yes | Yes | Yes | No | Not applicable | 6 |
| 5 | Torabi et al., 2018 | Yes | Yes | Yes | Yes | Yes | Yes | Yes | Yes | No | Not applicable | 8 |
| 6 | Ala’Ersheidat et al., 2019 | No | Yes | Unclear | No | Yes | Yes | Unclear | Yes | No | Not applicable | 4 |
| 7 | Zardawi et al., 2020 | No | Yes | Yes | No | Yes | Yes | Yes | Yes | No | Not applicable | 6 |
| 1. Abbreviations: JBI, Joanna Briggs Institute 2. Note: Q1 to Q10 indicate questions 1 to 10 based on the JBI critical appraisal checklist as following: 3. Q1-Were there clear criteria for inclusion in the case series? 4. Q2-Was the condition measured in a standard, reliable way for all participants included in the case series? 5. Q3-Were valid methods used for identification of the condition for all participants included in the case series? 6. Q4-Did the case series have consecutive inclusion of participants? 7. Q5-Did the case series have complete inclusion of participants? 8. Q6-Was there clear reporting of the demographics of the participants in the study? 9. Q7-Was there clear reporting of clinical information of the participants? 10. Q8-Were the outcomes or follow up results of cases clearly reported? 11. Q9-Was there clear reporting of the presenting site(s)/clinic(s) demographic information? 12. Q10-Was statistical analysis appropriate? 13. * The JBI checklist has following four responses: Yes, No, Unclear, Not applicable. For "Yes" will be equal to 1 point, and 0 point will be considered for other responses. | | | | | | | | | | | | |

| Table S4 (c). Critical appraisal for case-reports included in the systematic review according to JBI | | | | | | | | | | |
| --- | --- | --- | --- | --- | --- | --- | --- | --- | --- | --- |
| ID | Author, year | Q1 | Q2 | Q3 | Q4 | Q5 | Q6 | Q7 | Q8 | Total score^*^ |
| 1 | Rosenblatt et al., 2006 | Yes | Yes | Yes | Yes | Yes | Yes | Yes | Yes | 8 |
| 2 | Simon et al., 2007 | Yes | No | Yes | Yes | Yes | Yes | Yes | Yes | 7 |
| 3 | Gupta et al., 2010 | Yes | Yes | Yes | Yes | Yes | Yes | Yes | Yes | 8 |
| 4 | Humayun et al., 2010 | Yes | Yes | Yes | Yes | Yes | Yes | Yes | Yes | 8 |
| 5 | Sheth et al., 2013 | Yes | Yes | Yes | Yes | Yes | Yes | Yes | Yes | 8 |
| 6 | Vital Ribeiro-Júnior et al., 2013 | Yes | Yes | Yes | Yes | Yes | Yes | Yes | Yes | 8 |
| 7 | Gaddale et al., 2014 | Yes | Yes | Yes | Yes | Yes | Yes | Yes | Yes | 8 |
| 8 | Grover et al., 2014 | Yes | Yes | Yes | Yes | Yes | Yes | Yes | Yes | 8 |
| 9 | Pandurić et al., 2014 | Yes | Yes | Yes | Yes | Yes | Yes | Yes | Yes | 8 |
| 10 | Muthukumar et al., 2015 | Yes | Yes | Yes | Yes | Yes | Yes | Unclear | Yes | 7 |
| 11 | Sthapak et al., 2015 | Yes | No | Yes | Yes | Yes | Yes | No | Yes | 6 |
| 12 | Tasdemir et al., 2015 | Yes | Yes | Yes | Yes | Yes | Yes | Yes | Yes | 8 |
| 13 | Pawar et al., 2015 | Yes | Yes | Yes | Yes | Yes | No | No | Unclear | 5 |
| 14 | Funde et al., 2016 | Yes | No | Yes | Yes | Yes | Yes | Yes | Yes | 7 |
| 15 | Littuma, et al., 2017 | Yes | Yes | Yes | Yes | Yes | Yes | Yes | Yes | 8 |
| 16 | Moideen et al., 2017 | Yes | Yes | Yes | Yes | Yes | Yes | Yes | Yes | 8 |
| 17 | Ambrosio et al. 2018 | Yes | No | Yes | Yes | Yes | Yes | Yes | Yes | 7 |
| 18 | Mathew, 2018 | Yes | Yes | No | No | No | No | No | Yes | 3 |
| 19 | Gwi-Hyeon Min et al., 2018 | Yes | Yes | Yes | Yes | Yes | Yes | Yes | Yes | 8 |
| 20 | Foudah, 2019 | Yes | Yes | Yes | Yes | Yes | Yes | Yes | Yes | 8 |
| 21 | Gadalla et al., 2019 | Yes | Yes | Yes | Yes | Yes | Yes | Yes | Yes | 8 |
| 22 | Mohanty et al., 2019 | Yes | Yes | Yes | Yes | Yes | Yes | Yes | Yes | 8 |
| 23 | Thaker et al., 2019 | Yes | Yes | Yes | Yes | Yes | Yes | Yes | Yes | 8 |
| 24 | Iikura et al., 2019 | Yes | Yes | Yes | Yes | Yes | Yes | Yes | Yes | 8 |
| 25 | Duruel et al., 2020 | Yes | No | Yes | Yes | Yes | Yes | Yes | Yes | 7 |
| 26 | Sharma et al., 2020 | Yes | No | No | No | Yes | Yes | Yes | Yes | 5 |
| 1. Abbreviations: JBI, Joanna Briggs Institute 2. Note: Q1 to Q8 indicate questions 1 to 8 based on the JBI critical appraisal checklist as following: 3. Q1- Were patient’s demographic characteristics clearly described? 4. Q2- Was the patient’s history clearly described and presented as a timeline? 5. Q3- Was the current clinical condition of the patient on presentation clearly described? 6. Q4- Were diagnostic tests or assessment methods and the results clearly described? 7. Q5- Was the intervention(s) or treatment procedure(s) clearly described? 8. Q6- Was the post-intervention clinical condition clearly described? 9. Q7- Were adverse events (harms) or unanticipated events identified and described? 10. Q8- Does the case report provide takeaway lessons? 11. * The JBI checklist has following four responses: Yes, No, Unclear, Not applicable. For "Yes" will be equal to 1 point, and 0 point will be considered for other responses. | | | | | | | | | | |
